# LOX overexpression programming mediates the osteoclast mechanism of low peak bone mass in female offspring rats caused by pregnant dexamethasone exposure

**DOI:** 10.1186/s12964-023-01115-2

**Published:** 2023-04-24

**Authors:** Tao Jiang, Hao Xiao, Bin Li, Hangyuan He, Hui Wang, Liaobin Chen

**Affiliations:** 1 Division of Joint Surgery and Sports Medicine, Department of Orthopedic Surgery, Zhongnan Hospital of Wuhan University, Wuhan, 430071 China; 2 Department of Pharmacology, Wuhan University School of Basic Medical Sciences, Wuhan, 430071 China; 3 Hubei Provincial Key Laboratory of Developmentally Originated Disease, Wuhan, 430071 China

**Keywords:** Dexamethasone, Osteoclast, Low peak bone mass, Reactive oxygen species, Lysyl oxidase, Intrauterine programming

## Abstract

**Background:**

Osteoporosis is a degenerative disease characterized by reduced bone mass, with low peak bone mass being the predominant manifestation during development and having an intrauterine origin. Pregnant women at risk of preterm delivery are commonly treated with dexamethasone to promote fetal lung development. However, pregnant dexamethasone exposure (PDE) can lead to reduced peak bone mass and susceptibility to osteoporosis in offspring. In this study, we aimed to investigate the mechanism of PDE-induced low peak bone mass in female offspring from the perspective of altered osteoclast developmental programming.

**Methods:**

0.2 mg/kg.d dexamethasone was injected subcutaneously into rats on gestation days (GDs) 9–20. Some pregnant rats were killed at GD20 to remove fetal rat long bones, the rest were delivered naturally, and some adult offspring rats were given ice water swimming stimulation for two weeks.

**Results:**

The results showed that the fetal rat osteoclast development was inhibited in the PDE group compared with the control group. In contrast, the adult rat osteoclast function was hyperactivation with reduced peak bone mass. We further found that the promoter region methylation levels of lysyl oxidase (LOX) were decreased, the expression was increased, and the production of reactive oxygen species (ROS) was raised in PDE offspring rat long bone before and after birth. Combined in vivo and in vitro experiments, we confirmed that intrauterine dexamethasone promoted the expression and binding of the glucocorticoid receptor (GR) and estrogen receptor β (ERβ) in osteoclasts and mediated the decrease of LOX methylation level and increase of expression through upregulation of 10–11 translocator protein 3 (Tet3).

**Conclusions:**

Taken together, we confirm that dexamethasone causes osteoclast LOX hypomethylation and high expression through the GR/ERβ/Tet3 pathway, leading to elevated ROS production and that this intrauterine epigenetic programming effect can be carried over to postnatal mediating hyperactivation in osteoclast and reduced peak bone mass in adult offspring. This study provides an experimental basis for elucidating the mechanism of osteoclast-mediated intrauterine programming of low peak bone mass in female offspring of PDE and for exploring its early targets for prevention and treatment.

Video Abstract

**Supplementary Information:**

The online version contains supplementary material available at 10.1186/s12964-023-01115-2.

## Background

Osteoporosis is a skeletal disease that results in increased bone fragility and fracture risk due to reduced bone mass and microarchitectural destruction of bone tissue, with a significantly higher prevalence in women than in men [1]. Low peak bone mass is a bone pathology that fails to reach the genetic potential of peak bone mass due to abnormal bone development. Patients with osteoporosis often exhibit reduced peak bone mass in youth [2, 3]. Epidemiological investigations suggest that osteoporosis has an intrauterine developmental origin [4]. Osteoclasts are an important class of cells derived from the mononuclear macrophage lineage that perform bone resorption functions and are essential for bone growth and remodeling and maintaining skeletal structure and homeostasis throughout the life cycle [5, 6]. Hyperactivation of osteoclast function is a significant cause of postmenopausal estrogen-deficient osteoporosis and periprosthetic infection-induced inflammatory osteoporosis [7, 8]. However, how do osteoclasts change during fetal-derived low peak bone mass and osteoporosis development? Are osteoclasts involved in its pathogenesis? No relevant studies have been reported.

Dexamethasone is a synthetic glucocorticoid drug widely used clinically for preterm birth and preterm birth-related diseases because it can enter the fetus through the placental barrier to promote fetal lung maturation and effectively prevent the occurrence of neonatal respiratory distress syndrome and reduce the mortality of preterm infants [9, 10]. In recent years, epidemiological investigations and experimental animal studies have revealed that dexamethasone is developmentally toxic and that prenatal application of dexamethasone can cause intrauterine growth retardation (IUGR) and skeletal dysplasia [11, 12]. Previous studies have shown that pregnant dexamethasone exposure (PDE) leads to poor long-bone development, reduced postnatal peak bone mass, and susceptibility to adult osteoporosis in offspring rats, associated with poor differentiation of osteoblasts and vascular endothelial cells induced by intrauterine dexamethasone exposure [13, 14]. However, it is unclear how osteoclasts change during the development of PDE-induced osteoporosis susceptibility in adult offspring and whether they mediate the onset of reduced peak bone mass after birth.

Reactive oxygen species (ROS), as the basis of redox reactions in the organism’s biochemistry [15], are a common mechanism by which multiple adverse environments during pregnancy lead to fetal programming phenotypes and multi-organ developmental toxicity [16, 17]. Studies have shown that PDE can lead to increased mitochondrial oxidative stress and elevated ROS in postnatal cardiomyocytes of the offspring, which mediates the occurrence of cardiovascular disease [18]. However, we don’t know whether altered bone local ROS homeostasis is involved in PDE-induced abnormal osteoclast development and reduced peak bone mass. Recent studies have found that epigenetic modifications of ROS-generating genes are involved in altered tissue local ROS homeostasis, such as altered levels of p53 apoptosis effector related to PMP-22 (PERP) histone methylation in skeletal muscle cause local ROS overproduction [19]. Intrauterine programming is a process in which permanent changes in tissue morphology and function occur due to multiple injuries during the intrauterine period [20], and epigenetic mechanisms are involved in the occurrence of intrauterine programming. PDE has been found to regulate fetal developmentally relevant gene expression and metabolic alterations through epigenetic modifications that mediate susceptibility to diseases such as osteoarthritis [21] and epilepsy [22] in the offspring after birth. Then, whether the epigenetic mechanism of ROS production regulates low peak bone mass due to PDE in bone local osteoclasts should be explored and studied in depth.

In the present study, we used a stably established rat model of PDE [23] to observe the developmental alterations of osteoclasts and the occurrence of low peak bone mass in the offspring, to investigate whether abnormal bone local ROS mediated the formation of low peak bone mass, and to elucidate further the intrauterine programming mechanism of osteoclast-mediated reduction of peak bone mass in PDE-induced offspring in combination with in vitro knockdown experiments. This study provides essential experimental evidence and new academic perspectives on the pathogenesis of fetal-derived osteoporosis and offers new ideas for exploring its early therapeutic targets.

## Methods

### Chemicals and reagents

Dexamethasone was obtained from the Shuanghe Pharmaceutical Co. (Wuhan, China). Isoflurane was obtained from Baxter Healthcare Co. (Deerfield, IL, USA). Paraformaldehyde was obtained from Aspen Technology Inc. (Beijing, China). H_2_O_2_, RU486 for glucocorticoid receptor (GR) inhibitor, and tartrate-resistant acid phosphatase (TRAP) assay kit were purchased from Sigma-Aldrich (St. Louis, MO, USA). ROS assay kit, tempol for ROS remover, and macrophage colony-stimulating factor (M-CSF) for bone marrow-derived macrophages (BMMs) proliferation were purchased from MedCemExpress Co. (New Jersey, USA). Phosphate buffered saline (PBS) solution, erythrocyte lysis solution, radioimmunoprecipitation assay (RIPA) lysate, and phenylmethanesulfonyl fluoride (PMSF) were purchased from Servicebio Co. (Wuhan, China). Enhanced chemiluminescence substrate was purchased from PerkinElmer Co. (Boston, MA, USA). Polyvinylidene fluoride (PVDF) membranes were purchased from EMD Millipore Co. (Burlington, MA, USA). Bovine serum albumin (BSA) and Triton were purchased from Roche Co. (Shanghai, China). The 3-(4,5-dimethyltiazol-2-yl)-5-(3-carboxymethoxyphenyl)-2-(4-sulfophenyl)-2H-tetrazolium (MTS) assay kit was purchased from Promega Co. (Madison, Wisconsin, USA). The receptor activator of nuclear kappa-b ligand (RANKL) for BMMs differentiation was purchased from R&D systems (Minnesota, USA). The nucleus and cytoplasm protein extraction kit and F-actin assay kit were purchased from Beyotime Biotech Co., Ltd. (Shanghai, China). The dulbecco’s modified eagle medium: nutrient mixture F-12 (DMEM/F12) was purchased from HyClone Co. (Logan, UT, United States), and the fetal bovine serum was purchased from Gibco Co. (Detroit, MI, United States). Trizol was purchased from Invitrogen Co. (Carlsbad, CA, USA). Reverse transcription and real-time quantitative polymerase chain reaction (RT-qPCR) kits were purchased from Takara Biotech Co., Ltd. (Dalian, China). The SYBR Green dye and lipofectamine 3000 were purchased from Applied Biosystems through Thermo Fisher Scientific (Foster City, CA, USA). DNA purification kit was purchased from Tiangen Biotech Co. (Beijing, China). G Sepharose beads were purchased from Millipore Co. (New York, USA). The antibodies for GR (sc-376426) and estrogen receptor β (ERβ) (sc-53494) were purchased from Santa Cruz Biotech Co. (Santa Cruz, CA, USA). The antibodies for the nuclear factor of active T cells 1 (NFATc1) (A1539), protooncogene c-Fos (c-Fos) (A0236), cathepsin K (CtsK) (A1782), lysyl oxidase (LOX) (A7698), 10–11 translocation protein 3 (Tet3) (A7612), glyceraldehyde-3-phosphate dehydrogenase (GAPDH) (AC002), immunoglobulin G (IgG) (A19711), HRP goat anti-mouse IgG (AS003), HRP goat anti-rabbit IgG (AS014), FITC goat anti-rabbit IgG (AS011), and rabbit control IgG (AC005) were purchased from ABclonal Biotech Co., Ltd (Wuhan, China). The siRNA oligonucleotides against cell LOX were purchased from Shanghai GenePharma Co. Ltd. (Shanghai, China).

### Animal and treatment

Animal experiments were performed in the Center for Animal Experiments of Wuhan University (Wuhan, China), accredited by the Association for Assessment and Accreditation of Laboratory Animal Care International. All animal-experimental procedures were approved by and performed following the Guidelines for the Care and Use of Laboratory Animals of the Chinese Animal Welfare Committee. The Institutional Animal Care and Use Committee of Wuhan University Center for Animal Experiments approved the protocol (No. 201719). To reduce bias in animal experiments, we housed and treated the rats with one technician while different co-authors were in charge of bone sample collection and data analysis. The procedures and treatment methods in animal experiments are described in Fig. S1. This study focused on the pathogenesis of low peak bone mass in female offspring rats.

Specific pathogen-free (SPF) Wistar rats (with weights of 220 ± 20 g for females and 260 ± 20 g for males) were obtained from the Experimental Center of Hubei Medical Scientific Academy (No. 2017–2018, certification number: 42000600014526, Wuhan, China). After one week of acclimation, animals were bred and mated as previously described [23]. Pregnant rats were divided into control and PDE group and transferred to individual cages. From gestational day (GD) 9 to GD20, rats in the control and PDE groups were injected subcutaneously with 0.2 mg/kg.d dexamethasone or an equal volume of saline, respectively. On GD20, some pregnant rats were anesthetized under isoflurane and then sacrificed. Pregnant rats had litter sizes between 8 and 14 pups in each group were considered qualified. The female fetal rats were quickly removed and decapitated. Three whole hindlimbs were randomly collected for each group and fixed in 4% paraformaldehyde, paraffin-embedded, and sectioned. And remaining fetal long bone samples collected from littermates were pooled, immediately frozen in liquid nitrogen, and stored at -80 °C for further analyses. Another part of the pregnant rats (including the control and PDE group) gave birth naturally. Based on the human-to-rat dose conversion ratio (human: rat = 1:6.17) and the clinical dosing criteria for 0.05–0.2 mg/kg.d dexamethasone [24], the 0.2 mg/kg.d dexamethasone exposure used in this study is equivalent to 0.03 mg/kg.d population dose, which is lower than the clinical dose [25]. Therefore, the dose selections of dexamethasone in vivo experiments in this study are based on a specific basis and have certain clinical significance.

The female offspring rats were weaned and given a regular diet. Some were anesthetized with isoflurane at postnatal week (PW) 6 and then sacrificed. Long bone samples were collected. Half of the remaining female offspring rats were given chronic stress at PW10. In order to establish the chronic stress rat model used in this study, half of the rats in the control and PDE groups were randomly selected to swim in ice water, and the number of each group (both the control and PDE groups before and after chronic stress) was guaranteed to be about 12. At PW10-PW12, rats in the chronic stress groups were given ice water (the water temperature was 5–7 °C) swimming for 5 min every day for two weeks. After swimming every day, the rats were dried and kept warm. All rats were anesthetized with isoflurane at PW12 and then sacrificed. Long bone samples were collected and partially preserved in 70% ethanol for micro-computed tomography (micro-CT) assay. The other part was frozen in liquid nitrogen and stored at -80 °C for further analysis.

### Micro-CT

The hindlimb bones dissected from PW12 female offspring rats were fixed with 70% ethanol and then scanned and analyzed with a micro-CT system (VivaCT 40; Scanco, Basserdorf, Switzerland). To determine the subchondral bone mass indexed, including bone volume per tissue volume (BV/TV), trabecula number (Tb.N), trabecular thickness (Tb.Th) and trabecula separation (Tb.Sp), 0.5–2.5 mm under the cartilage was selected as the region of interest. Specimens were scanned at a 16.880 μm resolution with an exposure time of 400 ms. Meanwhile, we adjusted an electric voltage of 85 kV and a current of 200 μA to allow for maximum differentiation between these mineralized and non-mineralized tissues and a 0.1 mm-thick aluminum filter. After ROI standardization, images were converted to grayscale with a scale ranging from 0 to 255, selecting a minimum value of 80 and a maximum value of 120 in all groups. These values were determined based on visualization of the cancellous bone structure located in the ROI. Images of three-dimensional reconstruction in the medial compartment of the subchondral bone and parameters of the trabecular bone were obtained by the NRecon software.

### In vitro osteoclast culture and siRNA treatment

Fresh primary bone marrow-derived macrophages were extracted from the hindlimb bone of 4-week-old female rats. Briefly, under aseptic conditions, the long bone tissue of the rat hindlimb was cut, and the bone marrow cavity was rinsed with PBS until the cavity turned from red to white. The fluid was collected and centrifuged, and the supernatant was discarded. Cells were lysed with erythrocyte lysis solution for 10 min, centrifuged again, and then the supernatant was discarded. After washing the cells twice with PBS, the cells were resuspended with a completely supplemented medium and then inoculated into culture flasks. 24 h later, the flask liquid was collected, centrifuged, and the supernatant discarded to obtain the cells. Based on the pre-experiments, we selected 100 ng/ml M-CSF and 50 ng/ml RANKL to induce BMMs differentiation. Specifically, BMMs were obtained by culturing in a complete medium supplemented with 100 ng/ml M-CSF for 3 days. Then, we induced BMMs differentiation with 100 ng/ml M-CSF and 50 ng/ml RANKL for 4 days to obtain osteoclasts. Besides, we also used the same method to extract primary BMMs from the control and PDE groups of PW12 adult female offspring rats and cultured them with the same conditions to obtain osteoclasts. According to the manufacturer’s protocol, the siRNAs were transfected into BMMs using Lipofectamine 3000. We used LOX-siRNA at a concentration of 100 nM, and we transfected LOX-siRNA for 8 h after cell adherence. The knockout efficiency of LOX-siRNA is higher than 50%, and the results are shown in Fig. S2. The sequences of the LOX siRNA are presented in Table S1.

### Cell viability assay

MTS was used to assess cell viability. BMMs (3 × 10^4^ cells/well) were inoculated in 96-well plates and incubated with culture medium (including M-CSF) overnight. Dexamethasone (0, 20, 100, 500, and 2500 nM), tempol (0, 40, 200, 1000, and 5000 μM), and H_2_O_2_ (0, 2, 10, 50, and 250 μM) were added into the wells separately. MTS solution (20 μl/well) was added into each well 48 h later and incubated for 2 h at 37 °C. The effects of compounds on cells were measured by absorbance at 490 nm using a spectrophotometer (BioTek, USA), and the results are shown in Fig. S3. Based on fetal rat blood dexamethasone concentration (267 nM) in 0.2 mg/kg.d PDE rat model in a previous study [26], combined with our results of the MTS assay, we selected 20, 100, and 500 nM dexamethasone for subsequent in vitro experiments. Therefore, this study’s concentration selections of dexamethasone in vitro experiments are reasonable. In addition, we selected 1000 μM tempol and 2, 10, and 50 μM H_2_O_2_ for subsequent in vitro experiments based on the results of the MTS assay.

### TRAP staining

All hindlimb bone samples were fixed in 4% paraformaldehyde for 48 h, then decalcified with ethylene diamine tetraacetic acid (EDTA) (0.3 M) for 28 days, dehydrated, embedded with paraffin, and finally sectioned into 4 µm slices. TRAP staining method for tissue was as follows. Paraffin sections were first dewaxed with xylene and then dehydrated through graded ethanol of decreasing concentration (100%, 90%, 80%, 70%; 5 min/concentration, respectively), washing with PBS for 5 min (triplicate), then soaked in preheated PBS at 37 °C for 10 min, incubated the staining with the prepared TRAP working solution for 2 h at 37 °C, followed by washing with PBS 3 times. Hematoxylin staining was performed for 3 min and then washed with PBS. Differentiation and blue return with differentiation and blue return solution were carried out. Finally, the slices were rehydrated and sealed with a neutral resin. For cell trap staining, after removing the medium, the cells were fixed by adding 4% paraformaldehyde, and the cell membrane was broken by adding 0.1% Triton. After cleaning with PBS, the TRAP working solution was added. After incubation at 37 °C for 1 h, washing with PBS, redyeing with hematoxylin for 2 min, and washing with ddH_2_O, the cells were stained for TRAP enzymatic activity according to the TRAP staining kit scheme. NIS Elements BR light microscope (Nikon, Japan) was used for photography, and ImageJ software was used for quantification analyses. TRAP^+^ multinucleated (> 3 nuclei) cells were regarded as mature osteoclasts.

### F-actin staining

For cell F-actin staining, after removing the medium, the cells (6 × 10^4^ cells/well) were fixed by adding 4% paraformaldehyde for 30 min, and the cell membrane was broken by adding 0.1% Triton for 10 min. The cells were added F-actin fluorescent dye, followed by cleaning with PBS. After incubation at room temperature without light for 30 min and then washing with PBS, the cells were redyed with DAPI for 10 min without light. The cells were imaged using an inverted fluorescence microscope, and ImageJ software was used for quantification analyses.

### Detection of ROS levels

For detecting the intracellular ROS levels, according to the manufacturer’s protocol, ROS working solution containing medium was added into BMMs, which were incubated for 30 min at 37 °C protected from light. After that, the cells were washed twice with PBS, and then PBS was added for soaking. Images were collected using an inverted fluorescence microscope. The Image-Pro Plus 6.0 was used for quantification. Bone tissue ROS staining was performed by Baiqiandu Biotechnology Co. (Wuhan, China). The Analytical methods are consistent with cellular experiments.

### Immunofluorescence (IF) staining

For bone tissue IF staining, paraffin sections were first dewaxed to water, then antigen repair was performed in a microwave oven with EDTA antigen repair buffer (medium heating for 3 min, low heating for 10 min). After drawing circles around the tissue with a histochemical pen, 3% BSA (Roche, USA) was added dropwise to the section and blocked for 30 min. Anti-NFATc1, anti-c-Fos, anti-CtsK, and anti-GAPDH antibodies (diluted 1:100) were added and incubated overnight at 4 °C in a humid chamber. After washing with deionized water 5 times, FITC goat anti-rabbit or anti-mouse IgG (H + L) (diluted 1:5000) secondary antibodies were incubated in the dark at room temperature for 60 min. The sections were added DAPI and set in the dark for 10 min at room temperature, following washing 3 times with PBS, then rinsing with deionized water for 15 min. Images were collected using an inverted fluorescence microscope (Nikon, Japan). The Image-Pro Plus 6.0 (Media Cybernetics, Silver Spring, MD, USA) was used for quantification. BMMs were fixed with 4% paraformaldehyde under 4 °C for 15 min and added with a blocking solution (10% goat serum-PBS) for 2 h at room temperature. The subsequent treatment was referred to the protocols used for sections.

### Total RNA extraction, reverse transcription and RT-qPCR

Total RNA from BMMs was extracted utilizing Trizol reagent in accordance with the manufacturer’s protocol. The isolated RNA was collected and quantified by Nanodrop 3000 (USA). The cDNA was generated from RNA samples using M-MLV reverse transcriptase and oligo dT primers. RT-qPCR was performed using diluted cDNA. Total RNA from hindlimb bone tissues was extracted using the Trizol reagent. In brief, 30 mg of bone tissues were placed into a 1.5 ml EP tube with 2–3 zirconia beads and 1 ml of Trizol. The EP tubes were placed in a homogenizer (China; 60HZ, 4 min, 4 °C). After sufficient homogenization, the EP tube was added with 200 µl of chloroform, shaken vigorously for 30 s, and then placed on ice for about 15 min. After centrifugation (12,000 rpm, 4 °C, 15 min), the supernatants were carefully aspirated and transferred to a new EP tube with an equal volume of isopropanol, mixing the mixture upside down and then left at room temperature for 10 min. after centrifugation (12,000 rpm × 10 min, 4 °C), the supernatant was carefully discarded. After centrifugation (12,000 rpm × 10 min, 4 °C), the supernatant was carefully discarded, and the RNA precipitate was retained. The RNA was washed twice with pre-cooled 75% ethanol and mixed with the appropriate amount of ultrapure water. The isolated RNA generated cDNA, and the RT-qPCR procedure followed the above protocol. The PCR primers for amplification of rats, including NFATc1, c-Fos, acid phosphatase 5 (Acp5), CtsK, osteoclast-associated receptor (Oscar), dendritic cell-specific transmembrane protein (DC-stamp), GR, ERβ, DNA methyltransferase 1 (Dnmt1), Dnmt3a, Dnmt3b, Tet1, Tet2, Tet3, NADPH oxidases 1 (NOX1), NOX2, NOX4, NO synthase (NOS), LOX, cyclooxygenase1 (COX1), COX2, GAPDH, tyrosine 3-monooxygenase/tryptophan 5-monooxygenase activation protein, zeta (YWHAZ), succinate dehydrogenase (SDHA) and TATA box binding protein (TBP) are presented in Table S2. All primers were synthesized by Sangon Biotech (Shanghai, China). The relative amounts of the mRNA levels of the target genes were normalized to SDHA and TBP in fetal rat samples, GAPDH and YWHAZ in rat samples after birth, and GAPDH in vitro samples calculated by using the 2^−ΔΔCT^ method.

### Protein extraction and western blot analysis

Osteoclasts were collected with RIPA lysate containing 1% PMSF, and then proteins were extracted. The western blotting technique detected the corresponding protein expression in osteoclasts. After protein quantification, proteins were denatured, separated on sodium dodecyl sulfate–polyacrylamide gel electrophoresis (SDS-PAGE) gels, and transferred onto PVDF membranes. The membranes were immunoblotted with primary rabbit antibody for NFATc1, c-Fos, and CtsK, respectively (diluted 1:1000), and primary mouse antibody for GAPDH (diluted 1:5000) overnight at 4 °C. The next day, membranes were washed and then incubated with HRP goat anti-rabbit IgG or HRP goat anti-mouse IgG antibodies (diluted 1:5000) at room temperature for 1 h. Antibodies were detected with enhanced chemiluminescence substrate, and ImageJ software was used for quantification analyses.

### Chromatin immunoprecipitation (ChIP) assay

Pretreated long bone tissues and BMMs were chromatin cross-linked with the formaldehyde solution, and the addition of 125 mM glycine stopped the reaction. Samples were added to 1 ml of Lysis Buffer, and the lysates were sonicated for 4 min (2 s on, 2 s off, output power to 30%) and precleared with protein G Sepharose beads. Aliquots of the precleared, sheared chromatin were then immunoprecipitated using GR antibody. The samples were incubated overnight on the rocker at 4 °C, followed by fractional elution. The pellet was resuspended and set with proteinase K at 65 °C overnight and subsequently purified with the DNA purification kit according to the manufacturer’s instructions. The isolated DNA was used for RT-qPCR analysis. The primers used are listed in Table S3. The data obtained from RT-qPCR were used to calculate the abundance of protein-rich in the promoter of each gene using the following formula: IP/input = 2^Ct input DNA – Ct IP DNA^.

### Co-immunoprecipitation (Co-IP) assay

The Co-IP assay was performed to detect the interaction of GR with ERβ in BMMs. After washing with pre-cooling PBS, the cells were lysed in 1.2 mL lysis buffer at -80 °C for 30 min. The samples were centrifuged at 14,000 rpm at 4 °C for 15 min, and the supernatant was transferred to a new column. Then, the samples were divided into three parts: the first was used for input protein, the other two were performed using 1 μg of either a mock antibody IgG (diluted 1:100) as a control or a GR antibody (diluted 1:100) followed by incubation overnight at 4 °C with gentle shaking. Then, the samples were incubated with protein G magnetic beads at 4 °C for 6 h. After immunoprecipitation, the samples were washed with lysis buffer. After three washes, the retained proteins were mixed with 30 μL loading buffer at 100 °C for 10 min. The protein complexes and input protein were then detected by Western blotting.

### Solexa sequencing and bisulfite sequencing PCR

Solexa sequencing analysis and bisulfite sequencing PCR analysis were performed by Genergy (Shanghai, China). In this study, solexa sequencing analysis was measured by the Illumina Hiseq3000 sequencing platform, which was used to conduct high-throughput sequencing of samples in the single-ended 50 bp sequencing mode. The original data should be removed by primer and adaptor sequences, and the sequencing fragments should be inspected and screened by the quality and length of the bases to select the sequencing fragments with reliable quality finally. Fastx_toolkit (0.0.14) software was used to dynamically remove the joint sequence fragments and poor quality fragments from the 3’ end of the sequencing data. Seqtk (1.0-r82-dirty) and self-written R (3.x.x) scripts were used to conduct quality control analysis for the pre-processed data. Sequence alignment was performed using Bowtie (1.0.0). GO and KEGG were used self-written scripts. The default parameters were used in all software except for self-written scripts. Bisulfite sequencing PCR was measured by Illumina Hiseq/Nova seq platform. The specific primers were designed and amplified based on Methylation FastTarget V4.1 software and tested and quality-checked with standards. The qualified primers were mixed into a multiplex PCR primer panel for amplification and purification. Samples were processed according to the EZ DNA Methylation-Gold™ Kit protocol, and agarose gel electrophoresis was performed. Finally, quantification and up-sequencing were performed. Agilent 2100 Bioanalyzer first verified the fragment length distribution. Then high-throughput sequencing was performed on Illumina Hiseq/Nova seq platform with 2 × 150 bp double-end sequencing mode to obtain FastQ data.

### Statistical analysis

Data were analyzed using GraphPad Prism software (version 8.0, La Jolla, CA, USA). All data were expressed as mean ± standard error of the mean (S.E.M). The One-way ANOVA test was used for multi-group comparison, followed by the Dunnett t-test to determine whether the difference between the two groups was significant. A value of *P* < 0.05 was considered statistically significant.

## Results

### PDE caused low peak bone mass in female adult rats accompanied by hyperactivation of osteoclast function and exacerbated after chronic stress

**Fig. 1:**
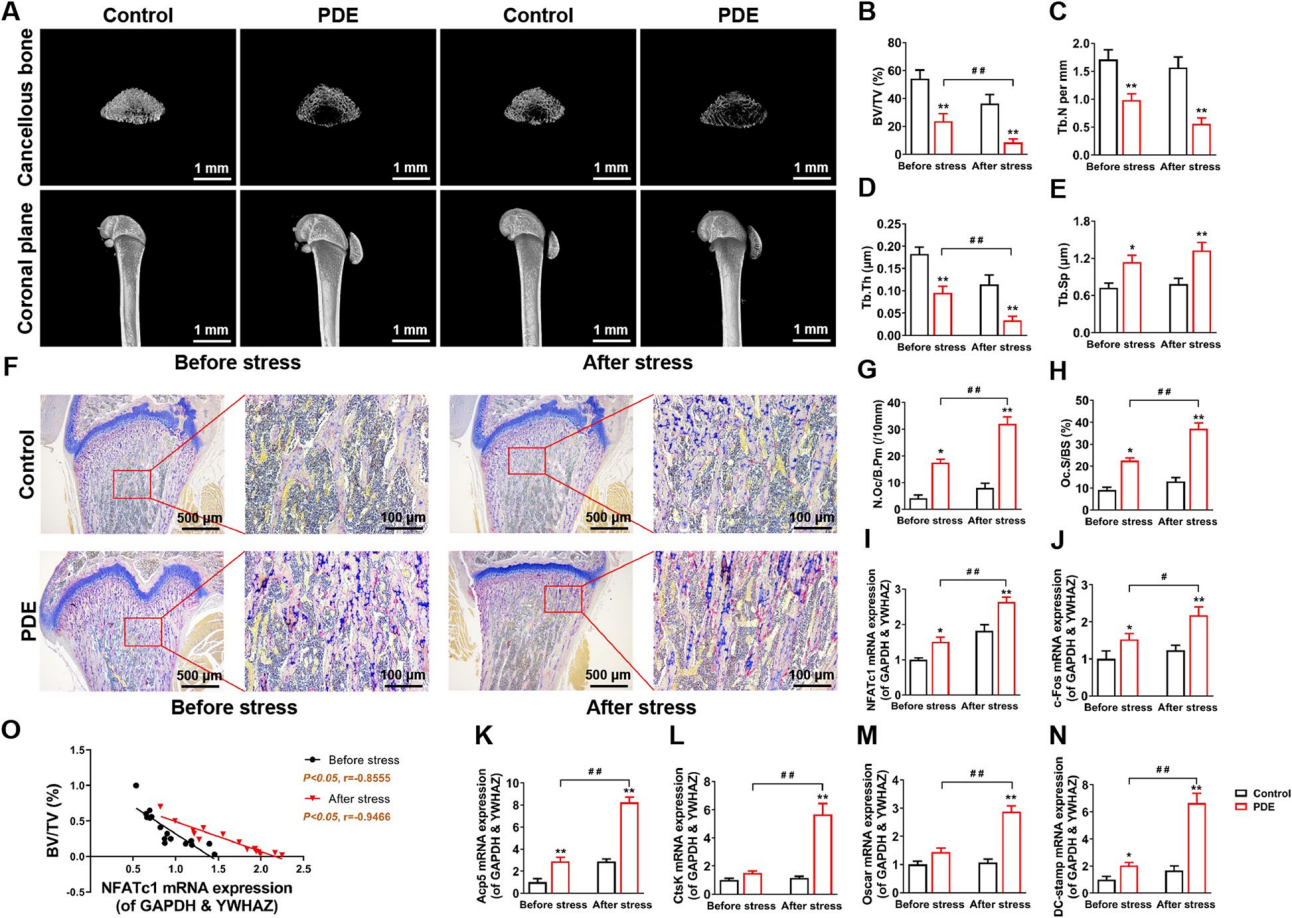
PDE induced low peak bone mass and hyperactivation of osteoclast function in female adult offspring rats. **A** Representative micro-CT images in each group of rats. **B**-**E** Quantification analyses of all bone sections, including BV/TV, Tb.N, Tb.Th, Tb.Sp. **F** Representative images of TRAP staining in decalcified bone sections of adult offspring rats. **G** Quantification analyses of N.Oc/B.Pm. **H** Quantification analyses of Oc.S/BS. **I**-**N** NFATc1, c-Fos, Acp5, CtsK, Oscar, and DC-stamp mRNA expression in bone tissue of adult offspring rats. **O** Correlation analysis between NFATc1 mRNA expression and BV/TV. Mean ± S.E.M., *n* = 8 per group for micro-CT, mRNA expression and correlation analysis, *n* = 3 per group for TRAP staining. ^*^P < 0.05, ^**^P < 0.01 *vs.* Control. ^#^*P* < 0.05, ^##^*P* < 0.01 *vs.* PDE. PDE: prenatal dexamethasone exposure; BV/TV: bone volume per tissue volume; Tb.N: trabecula number; Tb.Th: trabecular thickness; Tb.Sp: trabecula separation; TRAP: tartrate-resistant acid phosphatase; N.Oc/B.Pm: osteoclast number per bone perimeter; Oc.S/BS: osteoclast surface per bone surface; NFATc1: nuclear factor of active T cells 1; c-Fos: protooncogene c-Fos; Acp5: acid phosphatase 5; CtsK: cathepsin K; Oscar: osteoclast-associated receptor; DC-stamp: dendritic cell-specific transmembrane protein; GAPDH: glyceraldehyde-3-phosphate dehydrogenase; YWHAZ: tyrosine 3-monooxygenase/tryptophan 5-monooxygenase activation protein, zeta

First, we examined the bone mass of female adult offspring before and after chronic stress in PW12 by micro-CT. Compared with the control group, the bone mass of the PDE offspring was significantly lower (Fig. 1A), as evidenced by lower BV/TV, Tb.N, and Tb.Th, and higher Tb.Sp, and the bone mass of the PDE offspring was more significantly lower after chronic stress than before (*P* < 0.05, *P* < 0.01, Fig. 1B-E). TRAP staining and quantitative results showed that compared with the control group, the levels of osteoclast number per bone perimeter (N.Oc/B.Pm) and osteoclast surface per bone surface (Oc.S/BS) in the female trabecular bone of the PDE group were significantly increased (*P* < 0.05, *P* < 0.01, Fig. 1F-H), along with the mRNA expression of osteoclast marker genes NFATc1, c-Fos, Acp5, CtsK, Oscar, and DC-stamp significantly increased, and chronic stress aggravated osteoclast functional activation (*P* < 0.05, *P* < 0.01, Fig. 1I-N). In addition, there was a significant negative correlation between PDE-induced bone mass changes and osteoclast function before and after chronic stress (*P* < 0.05, Fig. 1O). The results of male offspring rats showed that the offspring osteoclast function was not significantly altered in the PDE group compared with the control group (Fig. S4). The above suggested a gender difference in osteoclast developmental alteration in PDE-induced offspring. The reduced peak bone mass in female adult offspring rats was related to the hyperactivation of osteoclast function, and the chronic stress aggravated the above alteration.

### PDE caused suppression of osteoclast function in intrauterine but no significant changes in the early postnatal period in female offspring rats

**Fig. 2:**
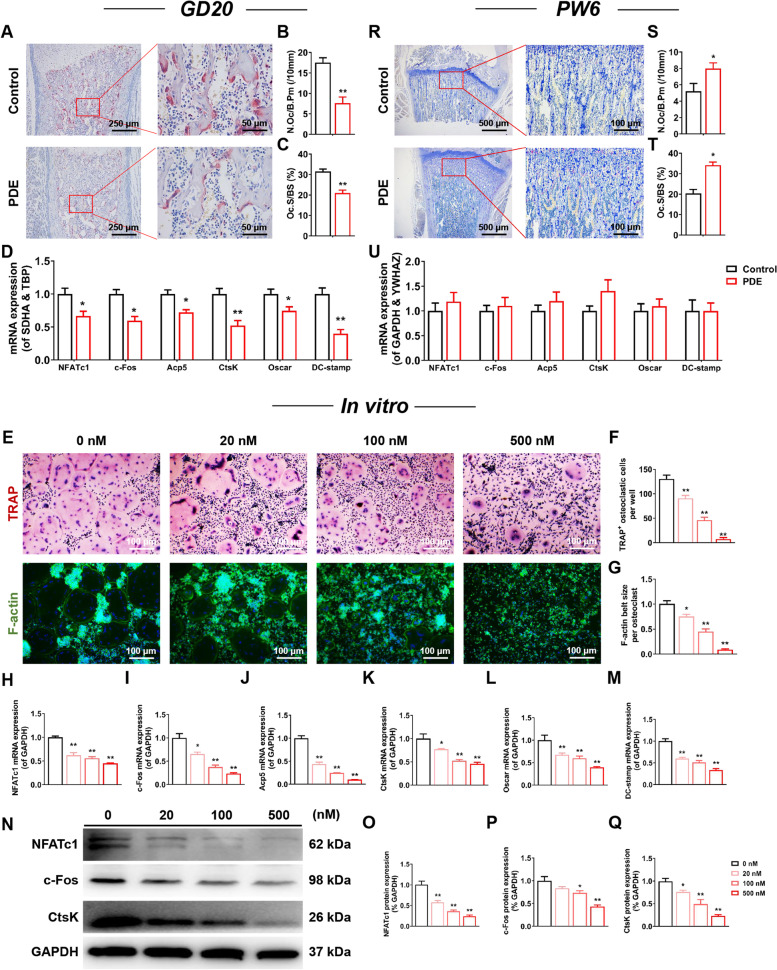
PDE induced inhibition of osteoclast function in fetal rats, while osteoclast function was not significantly altered in juvenile offspring rats. **A**, **R** Representative images of TRAP staining in decalcified bone sections of fetal rats and juvenile offspring rats. **B**, **S** Quantification analyses of N.Oc/B.Pm. **C**, **T** Quantification analyses of Oc.S/BS. **D**, **U** NFATc1, c-Fos, Acp5, CtsK, Oscar, and DC-stamp mRNA expression in bone tissue of fetal rats and juvenile offspring rats. **E** Representative images of TRAP and F-actin staining in osteoclasts treated with different concentrations of DEX. **F** Quantification analyses of TRAP^+^ osteoclastic cells number per well. **G** Quantification analyses of F-actin belt size per osteoclast. **H**-**M** NFATc1, c-Fos, Acp5, CtsK, Oscar, and DC-stamp mRNA expression in osteoclasts treated with different concentrations of DEX. **N** NFATc1, c-Fos, and CtsK protein expression in osteoclasts treated with different concentrations of DEX. **O**-**Q** Quantification analyses of NFATc1, c-Fos, and CtsK protein expression. Mean ± S.E.M., *n* = 3 per group for TRAP staining, F-actin staining, and western blotting, *n* = 8 per group for mRNA expression in vivo, *n* = 6 per group for mRNA expression in vitro. ^*^P < 0.05, ^**^P < 0.01 *vs.* Control in vivo or 0 nM DEX in vitro. PDE: prenatal dexamethasone exposure; TRAP: tartrate-resistant acid phosphatase; N.Oc/B.Pm: osteoclast number per bone perimeter; Oc.S/BS: osteoclast surface per bone surface; NFATc1: nuclear factor of active T cells 1; c-Fos: protooncogene c-Fos; Acp5: acid phosphatase 5; CtsK: cathepsin K; Oscar: osteoclast-associated receptor; DC-stamp: dendritic cell-specific transmembrane protein; DEX: dexamethasone; SDHA: succinate dehydrogenase; TBP: TATA box binding protein; GAPDH: glyceraldehyde-3-phosphate dehydrogenase; YWHAZ: tyrosine 3-monooxygenase/tryptophan 5-monooxygenase activation protein, zeta

Based on the altered osteoclast function and bone mass in female adult offspring rats due to PDE, we further traced the functional changes of osteoclasts in fetal rats to investigate the intrauterine origin of the phenomenon of low peak bone mass occurrence. In GD20, TRAP staining and quantification showed that N.Oc/B.Pm and Oc.S/BS were significantly lower in the long bones of fetal rats in the PDE group compared with the control group (*P* < 0.01, Fig. 2A-C), accompanied by mRNA expression of osteoclast marker genes NFATc1, c-Fos, Acp5, CtsK, Oscar, and DC-stamp were down-regulated (*P* < 0.05, *P* < 0.01, Fig. 2D). In vitro, after we treated BMMs that were extracted from the hind limb bone of 4-week-old female rats with different concentrations (20, 100, and 500 nM) of dexamethasone, TRAP staining, and quantification showed that dexamethasone inhibited osteoclast formation in a concentration-dependent manner, with the most pronounced inhibitory effect of 500 nM dexamethasone (*P* < 0.01, Fig. 2E, F). As shown in Fig. 2E, dexamethasone also significantly inhibited osteoclast F-actin ring formation, and its ring size decreased in a concentration-dependent manner by dexamethasone (*P* < 0.05, *P* < 0.01, Fig. 2G). In contrast the mRNA expression of osteoclast marker genes NFATc1, c-Fos, Acp5, CtsK, Oscar, and DC-stamp and protein expression of NFATc1, c-Fos, and CtsK were also downregulated in a concentration-dependent manner by dexamethasone (*P* < 0.05, *P* < 0.01, Fig. 2H-Q), a phenomenon consistent with the overall fetal mouse results (Fig. 2A-D). It was suggested that dexamethasone directly inhibited the formation and function of osteoclasts in female fetal rats.

Given the inconsistent alteration of osteoclast function in GD20 fetal rats and PW12 adult rats due to PDE, we performed relevant assays on PW6 juvenile offspring rats to investigate the pattern of PDE-induced alteration of osteoclast development. The results showed that N.Oc/B.Pm and Oc.S/BS were slightly elevated in the long bone tissue of juvenile offspring in the PDE group compared with the control group (*P* < 0.05, Fig. 2R-T). At the same time, there was no significant change in osteoclast marker gene expression (Fig. 2U). These results indicated that the osteoclast function in the early postnatal period in the offspring rats of the PDE group was comparable to that of the control group. It was suggested that the inhibition of osteoclast function originating from utero was alleviated in the early postnatal period and gradually transitioned to functional activation in adulthood.

### PDE caused increased ROS production before and after birth and further induced enhanced osteoclast function in female offspring rats

**Fig. 3:**
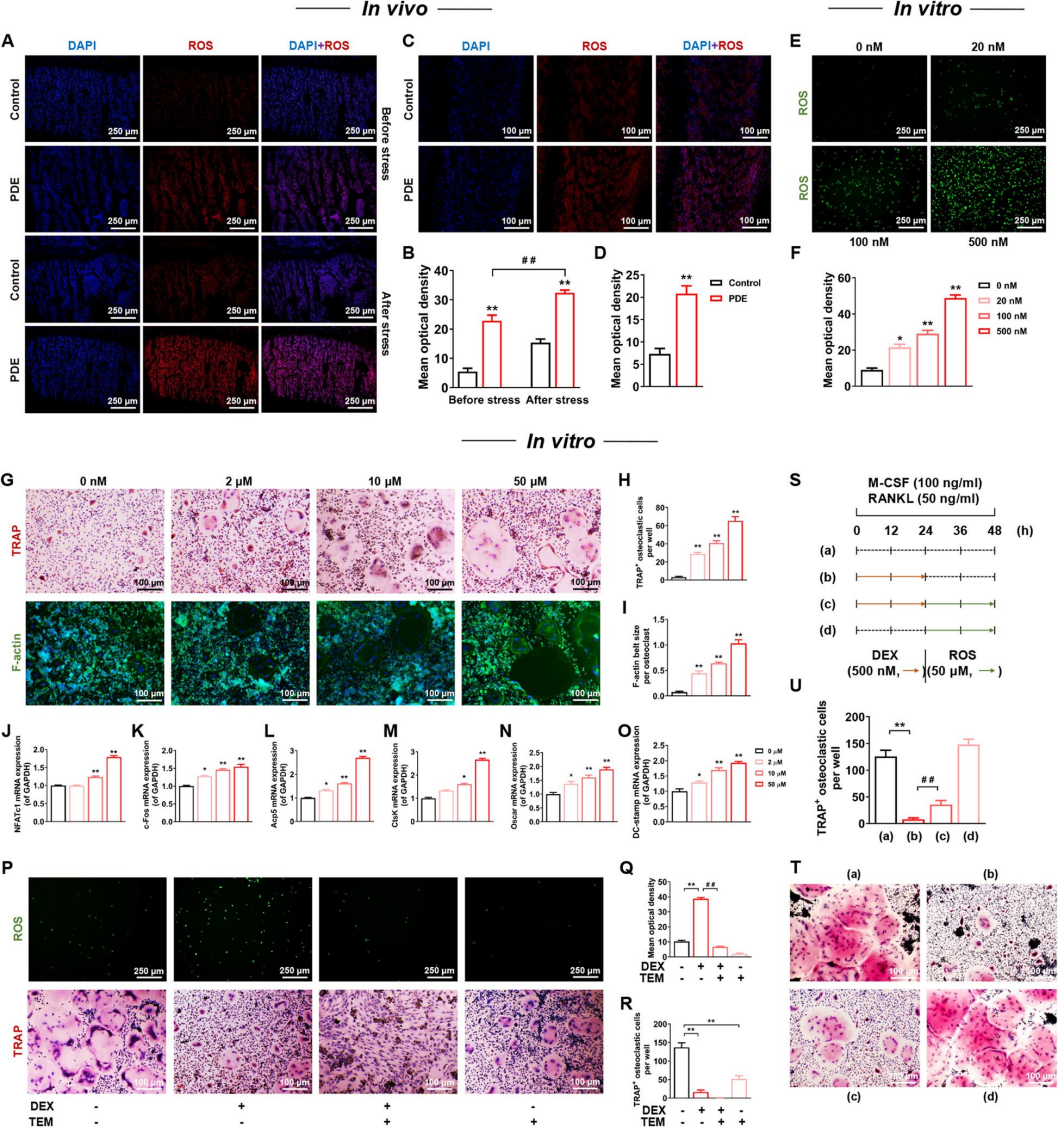
Elevated ROS in PDE offspring rats originated in the uterus and was of the potential to enhance osteoclast function. **A**, **C** Representative images of ROS production staining with DAPI in decalcified bone sections of fetal rats and adult offspring rats. **B**, **D** Quantification analyses of ROS mean optical density. **E** Representative images of ROS production staining in osteoclasts treated with different concentrations of DEX. **F** Quantification analyses of ROS mean optical density. **G** Representative images of TRAP and F-actin staining of osteoclasts treated with different concentrations of ROS. **H** Quantification analyses of TRAP^+^ osteoclastic cells number per well. **I** Quantification analyses of F-actin belt size per osteoclast. **J**-**O** NFATc1, c-Fos, Acp5, CtsK, Oscar, and DC-stamp mRNA expression in osteoclasts treated with different concentrations of ROS. **P** Representative images of ROS production and TRAP staining of osteoclasts in the 500 nM DEX group with or without TEM. **Q** Quantification analyses of ROS mean optical density. **R** Quantification analyses of TRAP^+^ osteoclastic cells number per well. **S** Experimental protocol for alternate treatment of osteoclasts with DEX and ROS. **T** Representative images of TRAP staining of osteoclasts treated with Fig. 4M protocol. **U** Quantification analyses of TRAP^+^ osteoclastic cells number per well. Mean ± S.E.M., *n* = 3 per group for ROS production, TRAP and F-actin staining, *n* = 6 per group for mRNA expression. ^*^P < 0.05, ^**^P < 0.01 *vs.* Control in vivo or 0 nM DEX in vitro. ^##^*P* < 0.01 *vs.* 500 nM DEX. ROS: reactive oxygen species; PDE: prenatal dexamethasone exposure; DEX: dexamethasone; TRAP: tartrate-resistant acid phosphatase; NFATc1: nuclear factor of active T cells 1; c-Fos: protooncogene c-Fos; Acp5: acid phosphatase 5; CtsK: cathepsin K; Oscar: osteoclast-associated receptor; DC-stamp: dendritic cell-specific transmembrane protein; DEX: dexamethasone; TEM: tempol; GAPDH: glyceraldehyde-3-phosphate dehydrogenase

Further, we explored PDE’s long-term effects and mechanisms on female offspring after birth. We found that ROS production in the long bones was significantly increased in the adult offspring rats of the PDE group compared with the control group by ROS fluorescent probe labeling and further elevated after chronic stress (*P* < 0.01, Fig. 3A, B). ROS alterations in long bone tissue of PDE fetal rats were consistent with those of adult rats (*P* < 0.01, Fig. 3C, D). In vitro, dexamethasone increased ROS levels in a concentration-dependent manner in BMMs (*P* < 0.01, Fig. 3E, F). It was suggested that PDE increased the production of long bone ROS in female offspring rats with an intrauterine origin.

To investigate the effect of ROS on osteoclast formation and function, we treated BMMs with different concentrations of ROS supplement H_2_O_2_. The results showed that ROS promoted osteoclast formation and function in a concentration-dependent manner, as evidenced by a significant increase in the number of TRAP^+^ osteoclasts and in the size of the F-actin ring (*P* < 0.01, Fig. 3G-I). Meanwhile, the mRNA expression of osteoclast marker genes NFATc1, c-Fos, Acp5, CtsK, Oscar, and DC-stamp increased with elevated ROS, and the most significant promoting effect was observed at 50 μM ROS (*P* < 0.05, *P* < 0.01, Fig. 3J-O). Next, we treated BMMs with ROS scavenger tempol and dexamethasone simultaneously. We found that tempol exacerbated the inhibitory effect of dexamethasone on osteoclast formation by TRAP staining (*P* < 0.01, Fig. 3P, R) based on ensuring the effective scavenging of ROS by tempol (*P* < 0.01, Fig. 3P, Q). Further, we designed an experiment to investigate the interaction between dexamethasone and ROS on osteoclasts, as shown in Fig. 3S. The TRAP staining showed that ROS supplementation after dexamethasone treatment could somewhat rescue the inhibitory effect of dexamethasone on osteoclast formation (*P* < 0.01, Fig. 3T, U). It was suggested that ROS could promote osteoclast formation and function enhancement and alleviate the osteoclast inhibitory effect induced by dexamethasone to a certain extent.

### PDE caused altered programming of LOX high expression in long bone tissue of female offspring rats

**Fig. 4:**
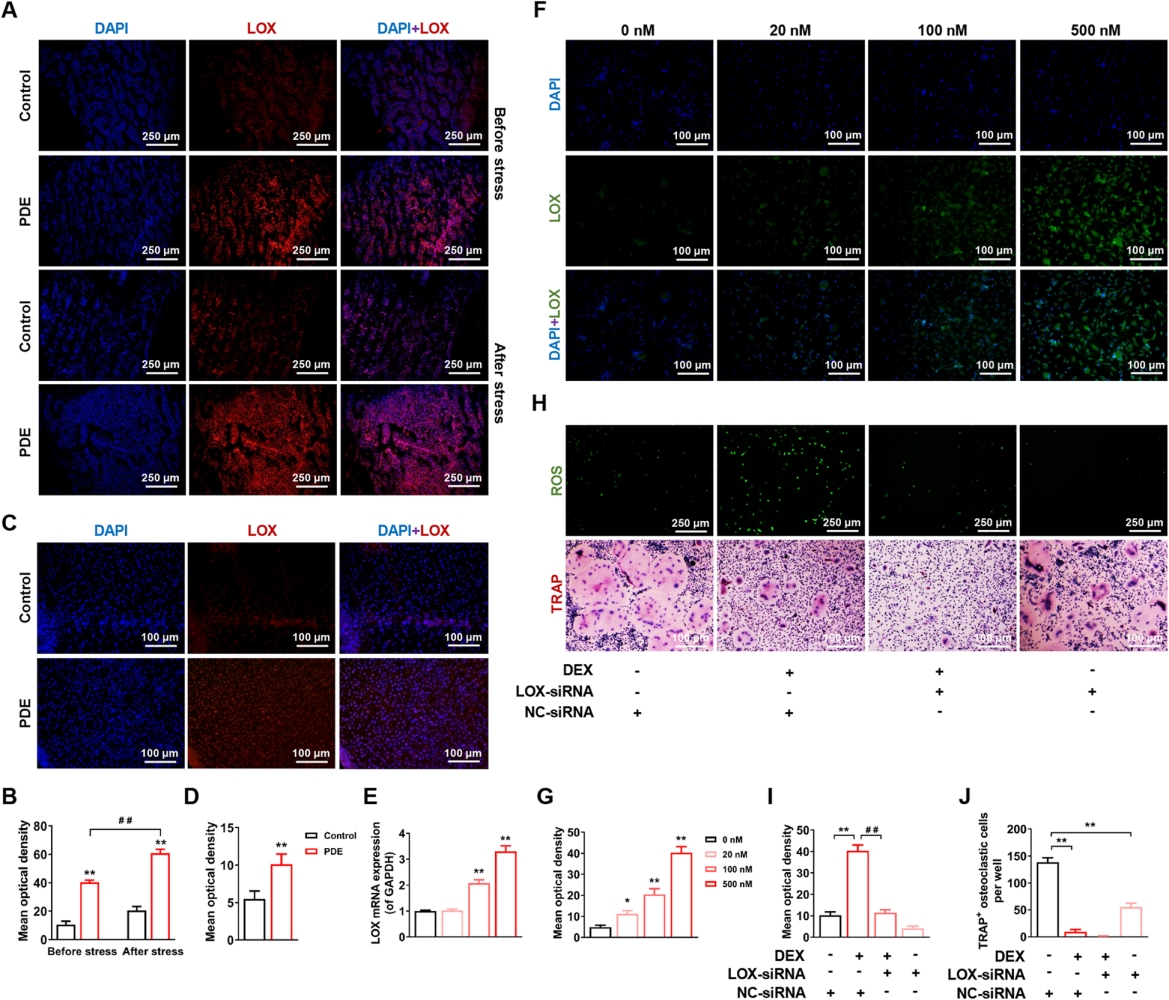
Programming of LOX high expression induced by PDE in offspring rats mediated elevated ROS. **A**, **C** Representative images of LOX immunohistochemical-paraffin staining with DAPI in decalcified bone sections of fetal and adult offspring rats. **B**, **D** Quantification analyses of LOX protein mean optical density. **E** LOX mRNA expression in osteoclasts treated with different concentrations of DEX. **F** Representative images of LOX immunohistochemical-paraffin staining with DAPI in osteoclasts treated with different concentrations of DEX. **G** Quantification analyses of LOX protein mean optical density. **H** Representative images of ROS production and TRAP staining of osteoclasts in the 500 nM DEX group with or without LOX-siRNA. **I** Quantification analyses of ROS mean optical density. **J** Quantification analyses of TRAP^+^ osteoclastic cells number per well. Mean ± S.E.M., *n* = 6 per group for mRNA expression in vitro, *n* = 3 per group for LOX immunohistochemical-paraffin, ROS production, and TRAP staining. ^*^P < 0.05, ^**^P < 0.01 *vs.* Control in vivo or 0 nM DEX in vitro. ^##^*P* < 0.01 *vs.* PDE in vivo or 500 nM DEX in vitro. LOX: lysyl oxidase; PDE: prenatal dexamethasone exposure; ROS: reactive oxygen species; TRAP: tartrate-resistant acid phosphatase; DEX: dexamethasone

To further investigate the ROS mechanism of PDE-induced altered osteoclast function, we performed transcriptome sequencing and validation by RT-qPCR technique. The results showed that the upregulation of LOX mRNA expression was consistent with the altered PDE-induced ROS elevation in female offspring rats’ long bone tissue (Fig. S5A-H). Further, IF staining of LOX protein in the long bones of PW12 adult rats and GD20 fetal rats showed that LOX protein expression was consistently elevated in the PDE group compared with the control group, and chronic stress could further increase its expression (*P* < 0.01, Fig. 4A-D). It was suggested that PDE could lead to altered programming of high LOX expression in female offspring.

In vitro, we confirmed that dexamethasone upregulated LOX mRNA and protein expression in a concentration-dependent manner (*P* < 0.05, *P* < 0.01, Fig. 4E-G), consistent with in vivo alterations. Further, we transfected BMMs with LOX-siRNA, and the results showed that the downregulation of LOX significantly reversed the dexamethasone-induced elevation of ROS (*P* < 0.05, *P* < 0.01, Fig. 4H, I). TRAP staining showed that downregulation of LOX further exacerbated the inhibitory effect of dexamethasone on osteoclast formation, and treatment with LOX-siRNA alone also inhibited ROS generation and osteoclast formation to some extent (*P* < 0.05, *P* < 0.01, Fig. 4H, J). It was suggested that dexamethasone mediated ROS elevation through the upregulation of LOX. Based on the above, we concluded that PDE mediated elevated ROS in female offspring through altered programming of osteoclast LOX high expression.

### PDE mediated hypomethylation of LOX promoter region in female offspring long bone tissue *via* GR/ERβ interactions

**Fig. 5:**
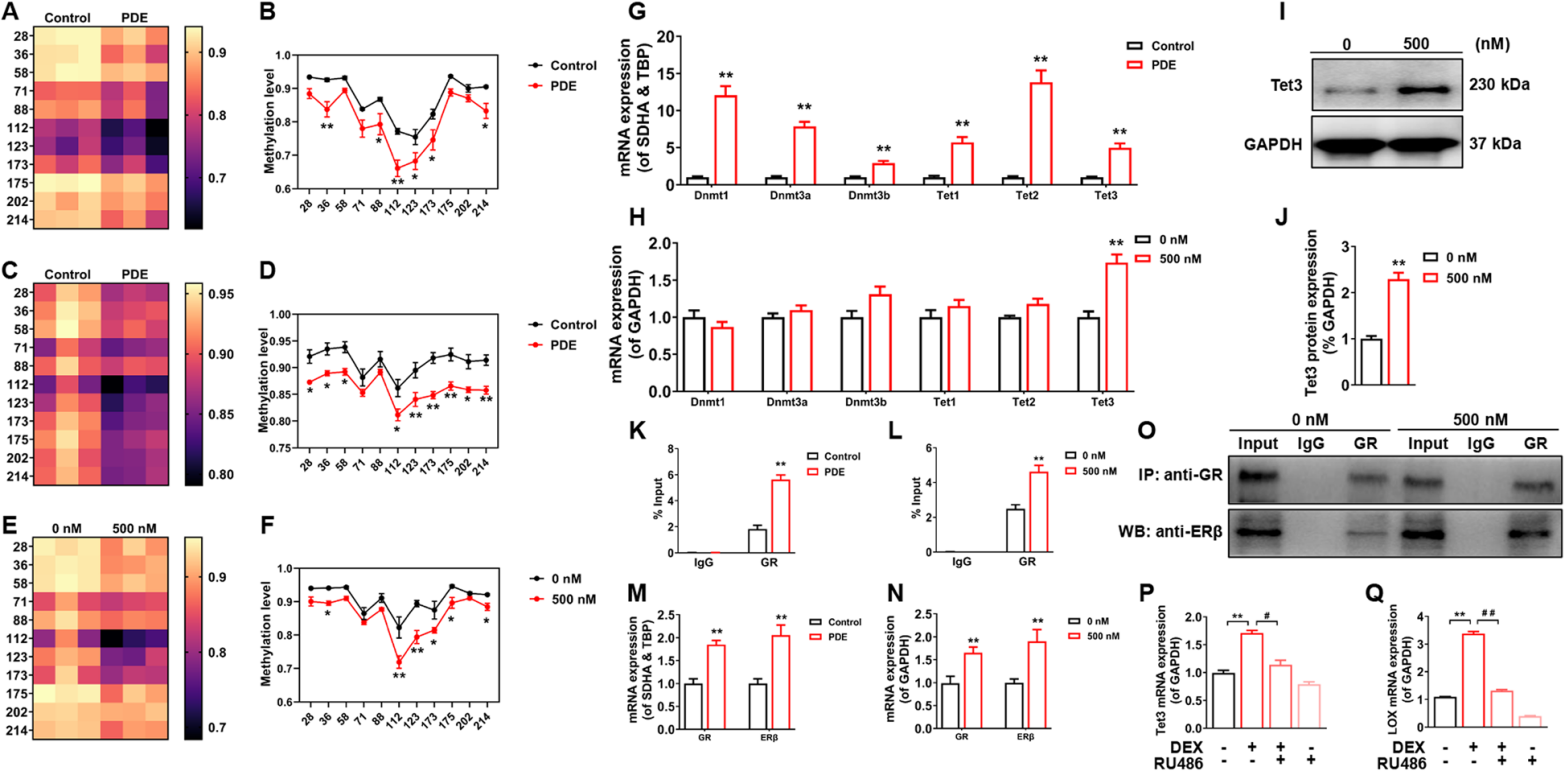
PDE induced the region hypermethylation change of the LOX promoter via GR and ERβ interactions. **A**-**F** Bisulfite Sequencing PCR of methylation levels in the LOX promoter region in bone tissue of fetal rats and adult offspring rats and osteoclasts treated with 500 nM DEX. **G**, **H** Screening of methyltransferases in bone tissue of fetal rats and osteoclasts treated with 500 nM DEX. **I** Tet3 protein expression in osteoclasts treated with 500 nM DEX. **J** Quantification analyses of Tet3 protein expression. **K**, **L** CHIP-PCR for the targeted binding of GR to LOX in bone tissue of fetal rats and osteoclasts treated with 500 nM DEX. **M**, **N** GR and ERβ mRNA expression in bone tissue of fetal rats and osteoclasts treated with 500 nM DEX. **O** Co-IP for GR and ERβ interactions in osteoclasts treated with 500 nM DEX. **P**, **Q** Tet3 and LOX mRNA expression in osteoclasts in the 500 nM DEX group with or without RU486. Mean ± S.E.M., *n* = 3 per group for Bisulfite Sequencing PCR, western blotting, ChIP-PCR, and Co-IP, *n* = 8 per group for mRNA expression in vivo, *n* = 6 per group for mRNA expression in vitro. ^*^P < 0.05, ^**^P < 0.01 *vs.* Control in vivo or 0 nM DEX in vitro. ^##^*P* < 0.01 *vs.* 500 nM DEX. PDE: prenatal dexamethasone exposure; LOX: lysyl oxidase; GR: glucocorticoid receptor; ERβ: estrogen receptor β; DEX: dexamethasone; Dnmt: DNA methyltransferase; Tet: ten-eleven translocation protein; SDHA: succinate dehydrogenase; TBP: TATA box binding protein; GAPDH: glyceraldehyde-3-phosphate dehydrogenase

Analysis of methylation sequencing results targeting the LOX promoter region at the animal level showed that the LOX promoter region was hypomethylated in the long bone tissue of female fetal and adult rats in the PDE group compared with the control group (*P* < 0.05, *P* < 0.01, Fig. 5A-D). In vitro, the methylation level of the LOX promoter region in osteoclasts was significantly reduced after 500 nM dexamethasone treatment (*P* < 0.05, *P* < 0.01, Fig. 5E, F), consistent with the in vivo changes. Then, we screened and validated the methylesterase and demethylase in vivo and in vitro. The results showed that in vivo, the mRNA expression of Dnmt1, Dnmt3a, Dnmt3b, Tet1, Tet2, and Tet3 were significantly upregulated in the long bones of fetal rats in the PDE group compared with the control group (*P* < 0.01, Fig. 5G); while in vitro, dexamethasone only upregulated Tet3 mRNA expression in osteoclasts (*P* < 0.01, Fig. 5H), and its protein level was altered consistent with the gene (Fig. 5I, J). It was suggested that Tet3 could be involved in dexamethasone-induced hypomethylation alterations in the LOX promoter region. Further, we predicted that glucocorticoid response elements (GRE) were present in the promoter region of LOX through the JASPAR website (https://jaspar.genereg.net/) (Fig S5I, J). Meanwhile, in vitro ChIP-PCR experiments confirmed that dexamethasone increased the binding of GR to the LOX promoter region (*P* < 0.01, Fig. 5K, L). It has been proved that, in female rats, GC promotes the expression of epigenetic modification enzymes through GR-ERβ interaction, thereby regulating downstream target gene epigenetic modification alterations [27]. We found that dexamethasone resulted in elevated mRNA expression of GR and ERβ in osteoclasts by in vivo and in vitro experiments (*P* < 0.01, Fig. 5M, N). Further, Co-IP experiments revealed that dexamethasone not only activated osteoclast GR but also increased the binding of GR and ERβ (Fig. 5O). In addition, the GR antagonist RU486 reversed the dexamethasone-induced high expression of Tet3 and LOX mRNA (*P* < 0.05, *P* < 0.01, Fig. 5P, Q). The above suggested that PDE mediated the altered hypomethylation of the LOX promoter region in female offspring through upregulation of Tet3 via GR/ERβ interactions.

### LOX-siRNA alleviated PDE-induced hyperactivation of primary osteoclast function in female adult offspring rats

**Fig. 6:**
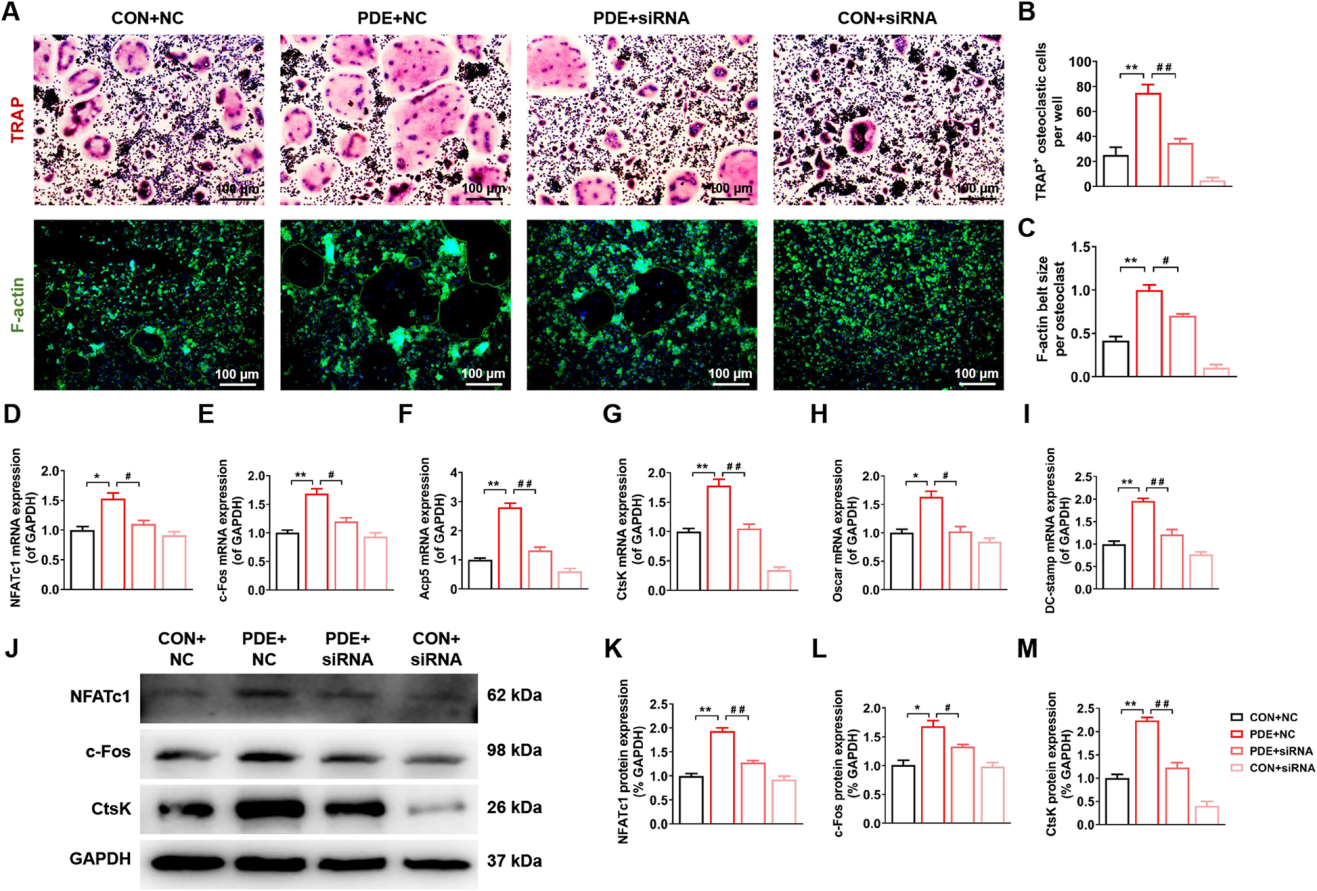
LOX-siRNA rescued hyperactivation of osteoclast function in female adult offspring rats induced by PDE. **A** Representative images of TRAP and F-actin staining of osteoclasts in the CON and PDE group with NC-siRNA or LOX-siRNA. **B** Quantification analyses of TRAP^+^ osteoclastic cells number per well. **C**: Quantification analyses of F-actin belt size per osteoclast. **D**-**I** NFATc1, c-Fos, Acp5, CtsK, Oscar, and DC-stamp mRNA expression in the CON and PDE group osteoclasts with NC-siRNA or LOX-siRNA. **J** NFATc1, c-Fos, and CtsK protein expression in osteoclasts in the CON and PDE group with NC-siRNA or LOX-siRNA. **K**-**M** Quantification analyses of NFATc1, c-Fos, and CtsK protein expression. Mean ± S.E.M., *n* = 3 per group for TRAP staining, F-actin staining and western blotting, *n* = 6 per group for mRNA expression. ^*^P < 0.05, ^**^P < 0.01 *vs.* CON + NC-siRNA. ^#^*P* < 0.05, ^##^*P* < 0.01 *vs.* PDE + NC-siRNA. PDE: prenatal dexamethasone exposure; TRAP: tartrate-resistant acid phosphatase; CON: control; NC-siRNA: negative control-small interfering RNA; NFATc1: nuclear factor of active T cells 1; c-Fos: protooncogene c-Fos; Acp5: acid phosphatase 5; CtsK: cathepsin K; Oscar: osteoclast-associated receptor; DC-stamp: dendritic cell-specific transmembrane protein; GAPDH: glyceraldehyde-3-phosphate dehydrogenase

To investigate whether inhibition of LOX could alleviate PDE-induced hyperactivation of osteoclast function, we extracted primary BMMs from the control and PDE groups of PW12 female adult rats and transfected them with NC-siRNA and LOX-siRNA, respectively. TRAP and F-actin staining results showed that compared with the PDE + NC-siRNA group, the PDE + LOX-siRNA group showed decreased osteoclast formation and function (*P* < 0.05, *P* < 0.01, Fig. 6A-C), accompanied by significant downregulation of mRNA expression of osteoclast marker genes NFATc1, c-Fos, Acp5, CtsK, Oscar, and DC-stamp and protein expression of NFATc1, c-Fos and CtsK (*P* < 0.05, *P* < 0.01, Fig. 6D-M). It was suggested that inhibition of LOX expression in BMMs could alleviate PDE-induced hyperactivation of osteoclast function in female adult offspring rats.

## Discussion

### PDE leads to reduced peak bone mass in female offspring with an intrauterine developmental origin

Dexamethasone is widely used clinically for preterm delivery and related diseases to promote fetal lung maturation and reduce neonatal morbidity and mortality [28, 29]. In pregnant women at 23–34 weeks of gestation with a tendency to preterm delivery, single or multiple courses of dexamethasone are routinely given to promote fetal lung maturation and reduce the incidence of neonatal respiratory distress syndrome [30]. However, a growing number of studies have shown that prenatal dexamethasone has a “double-edged sword” effect, effectively treating conditions such as prematurity while causing multiple organ developmental toxicity in the offspring, such as adrenal hypoplasia, neurodevelopmental impairment, and osteochondral dysplasia [31, 32]. Chronic stress (e.g., a 2-week ice water swimming experiment) is a typical means of inducing osteoporosis phenotype in rats. The degree of bone mass alteration before and after chronic stress can evaluate the susceptibility of rats to osteoporosis [33–35]. Peak bone mass is the maximum mineral density accumulated during growth and development and consolidated in early adulthood. In healthy girls, the rate of bone accumulation peaks between the ages of 11–14 years during puberty (equivalent to PW6-12 during rat development) and declines sharply approximately 2 years after menarche [36]. Thus, adolescence is a critical period for peak bone mass accumulation, and any reduction in peak bone mass caused by adverse factor stimuli can lead to susceptibility to osteoporosis in adulthood. The key to preventing osteoporosis is to establish adequate peak bone mass. In the present study, we observed that PDE caused a reduction in peak bone mass in female adult offspring and was more pronounced after chronic stress. This appropriately explained our previous finding [37] that PDE caused susceptibility to osteoporosis in female offspring and confirmed that PDE-induced low peak bone mass in female offspring had an intrauterine developmental origin.

### PDE-induced elevated ROS mediates excessive activation of osteoclast function and reduced peak bone mass in female offspring

Osteoclasts are important cells involved in bone remodeling and performing bone resorption functions. It has been found that the formation of peak bone mass is dependent on bone remodeling. When the balance of this tightly coupled process is disturbed (especially when osteoclast function is abnormally hyperactive), peak bone mass formation will be significantly reduced [38, 39]. In the present study, in vivo*,* and in vitro experiments confirmed that dexamethasone had a direct inhibitory effect on osteoclast formation and function, while the long-term impact induced by dexamethasone was to promote osteoclast formation and functional hyperactivation. Therefore, PDE caused osteoclast development in a pattern of “inhibition in utero and gradual enhancement after birth”. Studies have shown that PDE can cause IUGR, low birth weight, and catch-up growth in offspring rats [40–42]. The catch-up growth in body weight was reflected by the fact that PDE offspring had significantly lower birth weight, had almost the same weight as the control group in adolescence to adulthood, and had a significantly higher body weight growth rate than the control group throughout the developmental stage. It is similar to the “suppression followed by progressive enhancement” of osteoclasts in the female offspring due to PDE in this study. Therefore, we suggest that PDE-induced osteoclasts in female offspring show a developmental pattern similar to “catch-up growth”.

ROS, as one of the vital product forms of oxidative stress, is considered a causative factor for many diseases [43–45]. It has been found that heat stress early in life leads to increased oxidative stress and ROS in stickleback cells, causing catch-up growth and sperm DNA damage during the developmental stage, which mediates decreased survival and fertility in adulthood, even with multigenerational genetic effects [46]. Early postnatal ketamine exposure produces large amounts of ROS by inducing oxidative stress in the central nervous system of neonatal rats, which further affects immature neuronal development and has a long-term detrimental effect, mediating the development of schizophrenia in adulthood [47]. It was suggested that altered ROS homeostasis (especially elevated ROS) might regulate developmental abnormalities and disease development induced by exogenous damage. It has been shown that ROS is an important regulator of bone remodeling and that excess ROS disrupts bone remodeling homeostasis and mediates the reduction of bone mass by promoting osteoclast formation and function [48]. Based on the above, we hypothesized that a ROS-regulated mechanism might exist for the altered osteoclast development induced by PDE.

In the present study, in vivo experiments revealed that PDE induced increased bone local ROS production in the female offspring before and after birth; in vitro experiments confirmed that dexamethasone directly inhibited osteoclast formation and function on the one hand and induced increased osteoclast ROS production on the other. Unfortunately, ROS’s weak promotion of osteoclasts may not be sufficient to counteract the direct inhibitory effect caused by dexamethasone. Therefore, osteoclast development was inhibited during the intrauterine period, as evidenced by decreased formation and function. However, the inhibitory effect of dexamethasone on osteoclasts disappeared after the female offspring were removed from the environment of maternal dexamethasone exposure, while the upregulation of ROS by dexamethasone remained. We demonstrated that increased ROS production in PDE female offspring could continue to PW12 and accumulate further after chronic stress. Thus, persistently elevated ROS locally in bone promoted the development of osteoclasts in PDE female offspring and exhibited functional hyperactivation in adulthood, thereby mediating a decrease in peak bone mass.

### PDE upregulates Tet3 through GR/ERβ, which mediates hypomethylation programming of LOX and elevated ROS in osteoclasts

LOX is one of the key enzymes regulating ROS production, and overexpression of LOX significantly induces increased ROS synthesis in mammary epithelial cells and vascular smooth muscle cells [49, 50]. In the present study, we performed transcriptome sequencing of GD20 female fetal rat long bone tissue and screened seven genes associated with ROS generation. After validation by the RT-qPCR technique, we found that the mRNA expression of NOX2, LOX, and COX1 showed alterations consistent with elevated ROS. Next, we examined the long bone tissues of PW6 and PW12 offspring rats, and only LOX expression was consistent with the elevated changes in ROS, and combined with the in vitro results of NOX2 and COX1, the effect of both on ROS was excluded. Further, in vitro experiments also showed that dexamethasone-induced high expression of osteoclast LOX led to increased ROS production. In vitro,osteoclast LOX’s knockdown reversed the dexamethasone-induced increase in ROS production. It was suggested that osteoclast LOX was a key gene regulating the intrauterine dexamethasone-induced increase in local ROS production in female offspring bone. Moreover, we observed on PDE adult female offspring primary BMMs that the application of LOX-siRNA reversed the hyperactivation of osteoclast function induced by increased ROS production due to PDE. It was suggested that osteoclast LOX might be an early therapeutic target for fetal-originated osteoporosis induced by PDE.

Previously, several studies by our team demonstrated that glucocorticoids act on the GRE binding site in the promoter region of target genes via GR to regulate their expression alterations [51, 52]. And it has been shown that dexamethasone regulates LOX expression in fetal mouse lung tissue through GR targeting [53]. In this study, we predicted and found that GRE binding sites exist in the LOX promoter region through the JASPAR website. Further in vitro experiments confirmed that dexamethasone promoted GR binding to LOX and induced its expression upregulation and that the GR antagonist RU486 reversed the dexamethasone-induced LOX high expression. Thus, it was concluded that dexamethasone promoted LOX high expression in osteoclasts through GR targeting.

There is growing evidence that epigenetic modifications play an important role in the onset and development of fetal-derived disorders [54, 55]. DNA methylation, a common and relatively stable epigenetic modification, is considered a major regulatory mechanism for altered programming of fetal development [56, 57]. Studies have shown that intrauterine growth restriction due to malnutrition during pregnancy is significantly associated with placental wnt family member 2 (Wnt2) hypomethylation [58], and the change of liver insulin-like growth factor 2 (IGF2) hypomethylation mediates liver dysplasia due to pregnant low protein diet [59]. Dexamethasone application to the fetus during pregnancy can lead to epigenetic modifications and altered expression of developmental and metabolism-related genes, which plays an important role in developing and progressing fetal-derived diseases mediated by intrauterine programming mechanisms [60, 61]. Therefore, we examined the methylation levels of the LOX promoter region in vivo and in vitro experiments. It was found that PDE caused a sustained decrease in LOX promoter region methylation levels in the long bone tissue of female offspring before and after birth. At the same time cellular-level experiments confirmed that dexamethasone could cause LOX promoter region hypomethylation levels in osteoclasts. Further experimental results showed that TET3 was involved in dexamethasone-induced LOX promoter region hypomethylation changes in osteoclasts. The specific mechanism was that dexamethasone promoted GR/ERβ interactions by upregulating GR and ERβ expression in osteoclasts, thereby inducing elevated Tet3 expression, and that the GR antagonist RU486 reversed this alteration. From this, we concluded that dexamethasone mediates LOX hypomethylation programming in osteoclasts through the upregulation of Tet3 via GR/ERβ interactions.

### Sex differences in fetal-originated low peak bone mass in offspring induced by PDE

Sex differences are common in the phenotypic changes and pathogenesis of fetal-originated diseases [62, 63]. Significant sex differences have been found in PDE-induced multi-disease susceptibility in offspring. For example, serum bile acid levels were elevated in prenatal dexamethasone-treated male fetuses but not in female fetuses. Elevated total serum bile acid in male fetal rats and decreased in female fetal rats were observed in PDE rat model, which was attributed to the dysfunction of placental bile acid transporters [64]. Recent studies have identified sex-dependent epigenetic mechanisms involved in sex differences in adrenal developmental toxicity induced by PDE, i.e., intrauterine dexamethasone leads to sex differences in histone acetylation and expression levels of 11β-hydroxysteroid dehydrogenase type 2 (11β-HSD2) by decreasing GR binding to the androgen receptor (AR) in male offspring or increasing GR binding to ERβ in female offspring, the latter mediating sex differences in adrenal developmental toxicity. In this study, we also examined osteoclast function in male offspring and found that osteoclast function was not significantly changed in PDE male offspring before and after birth. Combined with previous findings that elevated ACE histone acetylation regulates bone marrow mesenchymal stem cells (BMSCs) osteogenic differentiation inhibition, which mediates the reduction in peak bone mass in male offspring due to PDE [37], we concluded that low peak bone mass in the PDE-induced offspring had significant sex differences in terms of main functional cells and the underlying mechanism. This provided an important theoretical basis for the subsequent investigation of sex differences in fetal-originated diseases.

### Conclusions

**Fig. 7:**
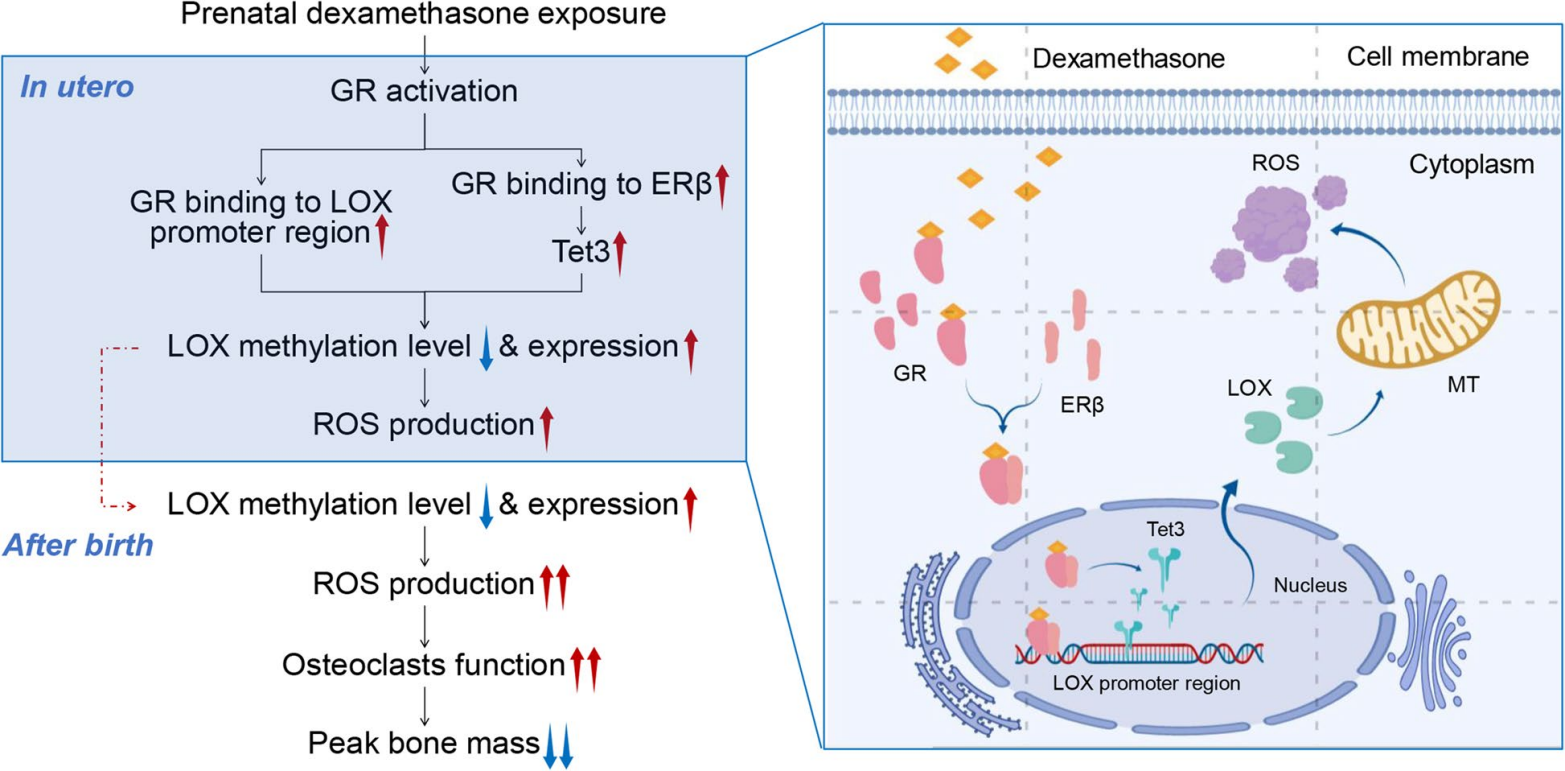
Intrauterine programming mechanism for the low peak bone mass in female offspring rats by PDE. PDE: prenatal dexamethasone exposure; DEX: dexamethasone; GR: glucocorticoid receptor; ERβ: estrogen receptor β; LOX: lysyl oxidase; Tet3: ten-eleven translocation protein 3; ROS: reactive oxygen species; osteoclasts: osteoclasts; MT: mitochondria

This study, for the first time, focused on the relationship between PDE-induced altered osteoclast developmental programming and low peak bone mass in female offspring and the regulatory mechanism and confirmed that PDE could lead to reduced peak bone mass in female adult offspring rats and elucidated the epigenetic mechanism of PDE-induced altered osteoclast development from the perspective of ROS regulation. That is, dexamethasone promotes the binding of osteoclast GR to the LOX promoter region by activating it on the one hand, leading to high LOX expression, and upregulates Tet3 by promoting GR binding to ERβ on the other hand, which in turn leads to hypomethylation levels and high expression of the LOX promoter region, causing increased ROS production. This programming effect mediated by abnormal epigenetic modifications can continue from intrauterine to postnatal and even adult life and is exacerbated after chronic stress, leading to persistently elevated ROS and osteoclast hyperactivation and ultimately mediating the occurrence of low peak bone mass in female offspring rats (Fig. 7). The low peak bone mass in PDE-induced offspring had significant sex differences in the mechanisms of occurrence and the above mechanism is not present in male offspring.

### Supplementary Information

**Additional file 1: Fig. S1.** The timetable and schematic procedure of animal treatment from gestational day (GD) 0 to postnatal week (PW) 12. **Fig. S2.** The experiments of the knockdown efficiency of LOX-siRNA on BMMs. (A, D): LOX mRNA expression in osteoclasts. (B, E): Representative images of LOX immunohistochemical-paraffin staining with DAPI in osteoclasts. (C, F): Quantification analyses of LOX protein mean optical density. Mean ± S.E.M., *n*=6 per group for mRNA expression, *n*=3 per group for LOX immunohistochemical-paraffin. ^**^P<1.01 *vs.* NC-siRNA in vitro or CON+NC in vivo. ^##^*P*<0.01 *vs.* DEX+NC-siRNA in vitro or PDE+NC in vivo. DEX: dexamethasone; LOX: lysyl oxidase; NC: negative control; CON: control; PDE: prenatal dexamethasone exposure. **Fig. S3.** Cell viability experiments on BMMs. (A) The cytotoxic effect of DEX on BMMs at different concentrations was measured by the MTS assay. (B) The cytotoxic effect of TEM on BMMs at different concentrations was measured by the MTS assay. (C) The cytotoxic effect of H_2_O_2_ on BMMs at different concentrations was measured by the MTS assay. Mean ± S.E.M., *n*=6 per group. ^**^P<0.01 *vs.* 0 group. DEX: dexamethasone; TEM: tempol; BMMs: bone marrow-derived macrophages; MTS: 3-(4,5-dimethyltiazol-2-yl)-5-(3-carboxymethoxyphenyl)-2-(4-sulfophenyl)-2H-tetrazolium. **Fig. S4.** PDE induced no significant change of osteoclast function in male offspring rats. (A, E): Representative images of TRAP staining in decalcified bone sections of fetal rats and adult offspring rats. (B, F): Quantification analyses of N.Oc/B.Pm. (C, G): Quantification analyses of Oc.S/BS. (D): NFATc1, c-Fos, Acp5, CtsK, Oscar and DC-stamp mRNA expression in bone tissue of fetal rats. (H-M): NFATc1, c-Fos, Acp5, CtsK, Oscar and DC-stamp mRNA expression in bone tissue of adult offspring rats. Mean ± S.E.M., *n*=3 per group for TRAP staining, *n*=8 per group for mRNA expression. ^**^P<0.01 *vs. *Control. ^##^*P*<0.01 *vs.* PDE. PDE: prenatal dexamethasone exposure; TRAP: tartrate resistant acid phosphatase; N.Oc/B.Pm: osteoclast number per bone perimeter; Oc.S/BS: osteoclast surface per bone surface; NFATc1: nuclear factor of active T cells 1; c-Fos: protooncogene c-Fos; Acp5: acid phosphatase 5; CtsK: cathepsin K; Oscar: osteoclast-associated receptor; DCstamp: dendritic cell specific transmembrane protein; SDHA: succinate dehydrogenase; TBP: TATA box binding protein; GAPDH: glyceraldehyde-3-phosphate dehydrogenase; YWHAZ: tyrosine 3- monooxygenase/tryptophan 5-monooxygenase activation protein, zeta. **Fig. S5.** Transcriptome sequencing results of female fetal rat long bone tissue and related experiments. (A, B): Transcriptome sequencing assays were performed in bone tissue of fetal rats. (C): ROS synthesis-related genes mRNA expression in bone tissue of fetal rats. (D): LOX mRNA expression in bone tissue of offspring rats. (E, F): NOX2 and COX1 mRNA expression in bone tissue of offspring rats. (G, H): NOX2 and COX1 mRNA expression in osteoclasts treated with different concentrations of DEX. (I, J): Predicted binding site of GR to LOX promoter region. Mean ± S.E.M., *n*=8 per group for mRNA expression in vivo, *n*=6 per group for mRNA expression in vitro. ^*^P<0.05, ^**^P<0.01 *vs.* Control in vivo or 0 nM DEX in vitro. LOX: lysyl oxidase; PDE: prenatal dexamethasone exposure; NOX: NADPH oxidases; NOS: NO synthase; COX: cyclooxygenase; SDHA: succinate dehydrogenase; TBP: TATA box binding protein; GAPDH: glyceraldehyde-3-phosphate dehydrogenase; YWHAZ: tyrosine 3-monooxygenase/tryptophan 5-monooxygenase activation protein, zeta; DEX: dexamethasone. **Table S1.** The LOX siRNA sequences used in BMMs transfection experiment. **Table S2.** Rat oligonucleotide primers and reaction conditions used in RT-qPCR. **Table S3.** Rat oligonucleotide primers and reaction conditions used in ChIP-PCR.

## Data Availability

The data that support the findings of this study are available from the corresponding author upon reasonable request.

## Abbreviations

IUGR: Intrauterine growth retardation
PDE: Pregnant dexamethasone exposure
ROS: Reactive oxygen species
PERP: P53 apoptosis effector related to PMP-22
GR: Glucocorticoid receptor
TRAP: Tartrate-resistant acid phosphatase
M-CSF: Macrophage colony-stimulating factor
BMMs: Bone marrow-derived macrophages
PBS: Phosphate buffered saline
RIPA: Radioimmunoprecipitation assay
PMSF: Phenylmethanesulfonyl fluoride
PVDF: Polyvinylidene fluoride
BSA: Bovine serum albumin
MTS: 3-(4,5-Dimethyltiazol-2-yl)-5-(3-carboxymethoxyphenyl)-2-(4-sulfophenyl)-2H-tetrazolium
RANKL: Receptor activator of nuclear kappa-b ligand
DMEM/F12: Dulbecco’s modified eagle medium: nutrient mixture F-12
RT-qPCR: Reverse transcription and real-time quantitative polymerase chain reaction
ERβ: Estrogen receptor β
NFATc1: Nuclear factor of active T cells 1
c-Fos: Protooncogene c-Fos
CtsK: Cathepsin K
LOX: Lysyl oxidase
Tet3: 10–11 Translocation protein 3
GAPDH: Glyceraldehyde-3-phosphate dehydrogenase
IgG: Immunoglobulin G
SPF: Specific pathogen-free
GD: Gestational day
PW: Postnatal week
micro-CT: Micro-computed tomography
BV/TV: Bone volume per tissue volume
Tb.N: Trabecula number
Tb.Th: Trabecular thickness
Tb.Sp: Trabecula separation
EDTA: Ethylene diamine tetraacetic acid
IF: Immunofluorescence
Acp5: Acid phosphatase 5
Oscar: Osteoclast-associated receptor
DC-stamp: Dendritic cell-specific transmembrane protein
Dnmt: DNA methyltransferase
NOX: NADPH oxidases
NOS: NO synthase
COX: Cyclooxygenase
TBP: TATA box binding protein
YWHAZ: Tyrosine 3-monooxygenase/tryptophan 5-monooxygenase activation protein, zeta
SDHA: Succinate dehydrogenase
SDS-PAGE: Sodium dodecyl sulfate–polyacrylamide gel electrophoresis
ChIP: Chromatin immunoprecipitation
Co-IP: Co-immunoprecipitation
SEM: Standard error of the mean
N.Oc/B.Pm: Osteoclast number per bone perimeter
Oc.S/BS: Osteoclast surface per bone surface
GRE: Glucocorticoid response elements
Wnt2: Wnt family member 2
IGF2: Insulin-like growth factor 2
11β-HSD2: 11β-Hydroxysteroid dehydrogenase type 2
AR: Androgen receptor
BMSCs: Bone marrow mesenchymal stem cells
