# Correction: LOX overexpression programming mediates the osteoclast mechanism of low peak bone mass in female offspring rats caused by pregnant dexamethasone exposure

**DOI:** 10.1186/s12964-024-01579-w

**Published:** 2024-03-26

**Authors:** Tao Jiang, Hao Xiao, Bin Li, Hangyuan He, Hui Wang, Liaobin Chen

**Affiliations:** 1 Division of Joint Surgery and Sports Medicine, Department of Orthopedic Surgery, Zhongnan Hospital of Wuhan University, Wuhan, 430071 China; 2 Department of Pharmacology, Wuhan University School of Basic Medical Sciences, Wuhan, 430071 China; 3 Hubei Provincial Key Laboratory of Developmentally Originated Disease, Wuhan, 430071 China

**Correction** **:** **Cell Commun Signal 21, 84 (2023)**

**https://doi.org/10.1186/s12964-023-01115-2**

Following publication of the original article [1], the authors identified an error in the Fig. 1F. In the published version, the image circled in red in Fig. 1F was misplaced since it didn't belong to the indicated group.

Published, incorrect version of Fig. 1F:

**Fig.1:**
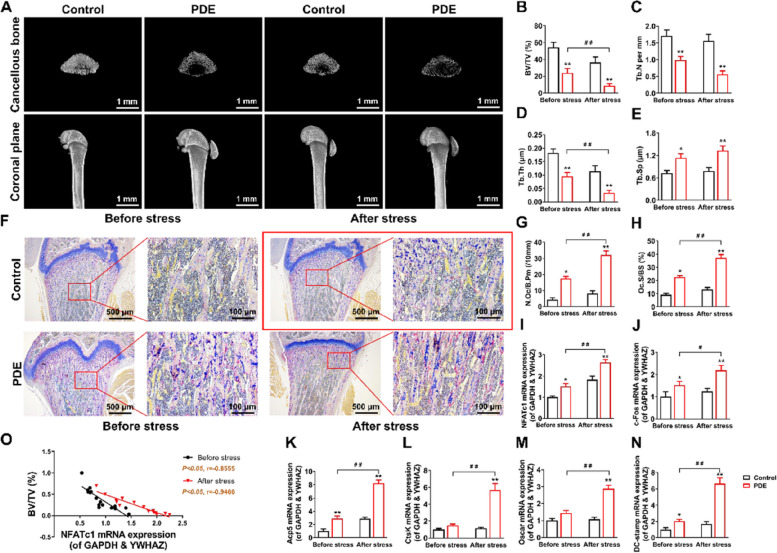
**(uncorrected)** PDE induced low peak bone mass and hyperactivation of osteoclast function in female adult offspring rats. **A** Representative micro-CT images in each group of rats. **B**-**E** Quantification analyses of all bone sections, including BV/TV, Tb.N, Tb.Th, Tb.Sp. **F** Representative images of TRAP staining in decalcified bone sections of adult offspring rats. **G** Quantification analyses of N.Oc/B.Pm. **H** Quantification analyses of Oc.S/BS. **I**-**N** NFATc1, c-Fos, Acp5, CtsK, Oscar, and DC-stamp mRNA expression in bone tissue of adult offspring rats. **O** Correlation analysis between NFATc1 mRNA expression and BV/TV. Mean ± S.E.M., *n* = 8 per group for micro-CT, mRNA expression and correlation analysis, *n* = 3 per group for TRAP staining. ^*^P < 0.05, ^**^P < 0.01 *vs.* Control. ^#^*P* < 0.05, ^##^*P* < 0.01 *vs.* PDE. PDE: prenatal dexamethasone exposure; BV/TV: bone volume per tissue volume; Tb.N: trabecula number; Tb.Th: trabecular thickness; Tb.Sp: trabecula separation; TRAP: tartrate-resistant acid phosphatase; N.Oc/B.Pm: osteoclast number per bone perimeter; Oc.S/BS: osteoclast surface per bone surface; NFATc1: nuclear factor of active T cells 1; c-Fos: protooncogene c-Fos; Acp5: acid phosphatase 5; CtsK: cathepsin K; Oscar: osteoclast-associated receptor; DC-stamp: dendritic cell-specific transmembrane protein; GAPDH: glyceraldehyde-3-phosphate dehydrogenase; YWHAZ: tyrosine 3-monooxygenase/tryptophan 5-monooxygenase activation protein, zeta

The authors have carefully rechecked our data records and have replaced it with the correct one from the raw data accordingly. Corrected Fig. 1F:

**Fig.1:**
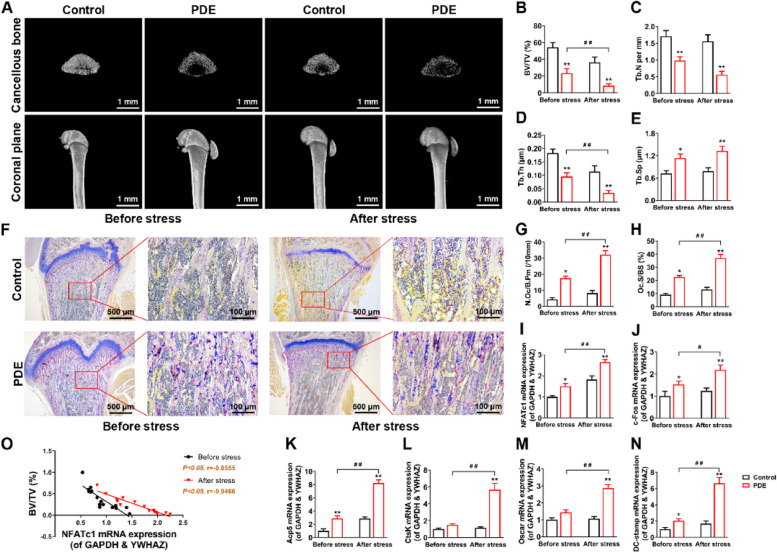
**(corrected)** PDE induced low peak bone mass and hyperactivation of osteoclast function in female adult offspring rats. **A** Representative micro-CT images in each group of rats. **B**-**E** Quantification analyses of all bone sections, including BV/TV, Tb.N, Tb.Th, Tb.Sp. **F** Representative images of TRAP staining in decalcified bone sections of adult offspring rats. **G** Quantification analyses of N.Oc/B.Pm. **H** Quantification analyses of Oc.S/BS. **I**-**N** NFATc1, c-Fos, Acp5, CtsK, Oscar, and DC-stamp mRNA expression in bone tissue of adult offspring rats. **O** Correlation analysis between NFATc1 mRNA expression and BV/TV. Mean ± S.E.M., *n* = 8 per group for micro-CT, mRNA expression and correlation analysis, *n* = 3 per group for TRAP staining. ^*^P < 0.05, ^**^P < 0.01 *vs.* Control. ^#^*P* < 0.05, ^##^*P* < 0.01 *vs.* PDE. PDE: prenatal dexamethasone exposure; BV/TV: bone volume per tissue volume; Tb.N: trabecula number; Tb.Th: trabecular thickness; Tb.Sp: trabecula separation; TRAP: tartrate-resistant acid phosphatase; N.Oc/B.Pm: osteoclast number per bone perimeter; Oc.S/BS: osteoclast surface per bone surface; NFATc1: nuclear factor of active T cells 1; c-Fos: protooncogene c-Fos; Acp5: acid phosphatase 5; CtsK: cathepsin K; Oscar: osteoclast-associated receptor; DC-stamp: dendritic cell-specific transmembrane protein; GAPDH: glyceraldehyde-3-phosphate dehydrogenase; YWHAZ: tyrosine 3-monooxygenase/tryptophan 5-monooxygenase activation protein, zeta

The authors declare that this correction will not affect any results and conclusions in this study.
