# Supplementary material for: LOX overexpression programming mediates the osteoclast mechanism of low peak bone mass in female offspring rats caused by pregnant dexamethasone exposure
Source: Cell Commun Signal. 2023 Apr 24;21:84. doi: 10.1186/s12964-023-01115-2 (PMC10124047; doi:10.1186/s12964-023-01115-2)
Supplement: Supplementary file 2 — Additional file 1: Fig. S1. The timetable and schematic procedure of animal treatment from gestational day (GD) 0 to postnatal week (PW) 12. Fig. S2. The experiments of the knockdown efficiency of LOX-siRNA on BMMs. (A, D): LOX mRNA expression in osteoclasts. (B, E): Representative images of LOX immunohistochemical-paraffin staining with DAPI in osteoclasts. (C, F): Quantification analyses of LOX protein mean optical density. Mean ± S.E.M., n=6 per group for mRNA expression, n=3 per group for LOX immunohistochemical-paraffin. **P<1.01 vs. NC-siRNA in vitro or CON+NC in vivo. ##P<0.01 vs. DEX+NC-siRNA in vitro or PDE+NC in vivo. DEX: dexamethasone; LOX: lysyl oxidase; NC: negative control; CON: control; PDE: prenatal dexamethasone exposure. Fig. S3. Cell viability experiments on BMMs. (A) The cytotoxic effect of DEX on BMMs at different concentrations was measured by the MTS assay. (B) The cytotoxic effect of TEM on BMMs at different concentrations was measured by the MTS assay. (C) The cytotoxic effect of H2O2 on BMMs at different concentrations was measured by the MTS assay. Mean ± S.E.M., n=6 per group. **P<0.01 vs. 0 group. DEX: dexamethasone; TEM: tempol; BMMs: bone marrow-derived macrophages; MTS: 3-(4,5-dimethyltiazol-2-yl)-5-(3-carboxymethoxyphenyl)-2-(4-sulfophenyl)-2H-tetrazolium. Fig. S4. PDE induced no significant change of osteoclast function in male offspring rats. (A, E): Representative images of TRAP staining in decalcified bone sections of fetal rats and adult offspring rats. (B, F): Quantification analyses of N.Oc/B.Pm. (C, G): Quantification analyses of Oc.S/BS. (D): NFATc1, c-Fos, Acp5, CtsK, Oscar and DC-stamp mRNA expression in bone tissue of fetal rats. (H-M): NFATc1, c-Fos, Acp5, CtsK, Oscar and DC-stamp mRNA expression in bone tissue of adult offspring rats. Mean ± S.E.M., n=3 per group for TRAP staining, n=8 per group for mRNA expression. **P<0.01 vs. Control. ##P<0.01 vs. PDE. PDE: prenatal dexamethasone exposure; TRAP: tartrate resistant a [file 12964_2023_1115_MOESM1_ESM.pdf]

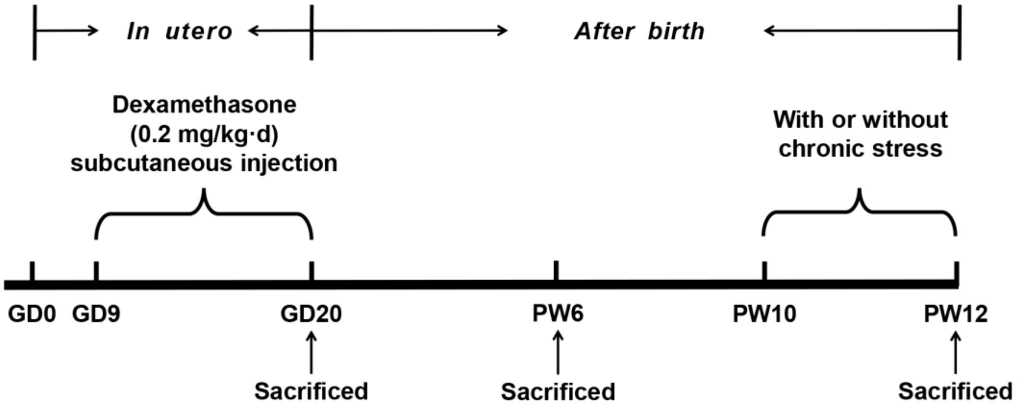

Fig S1. The timetable and schematic procedure of animal treatment from gestational day (GD) 0 to postnatal week (PW) 12.

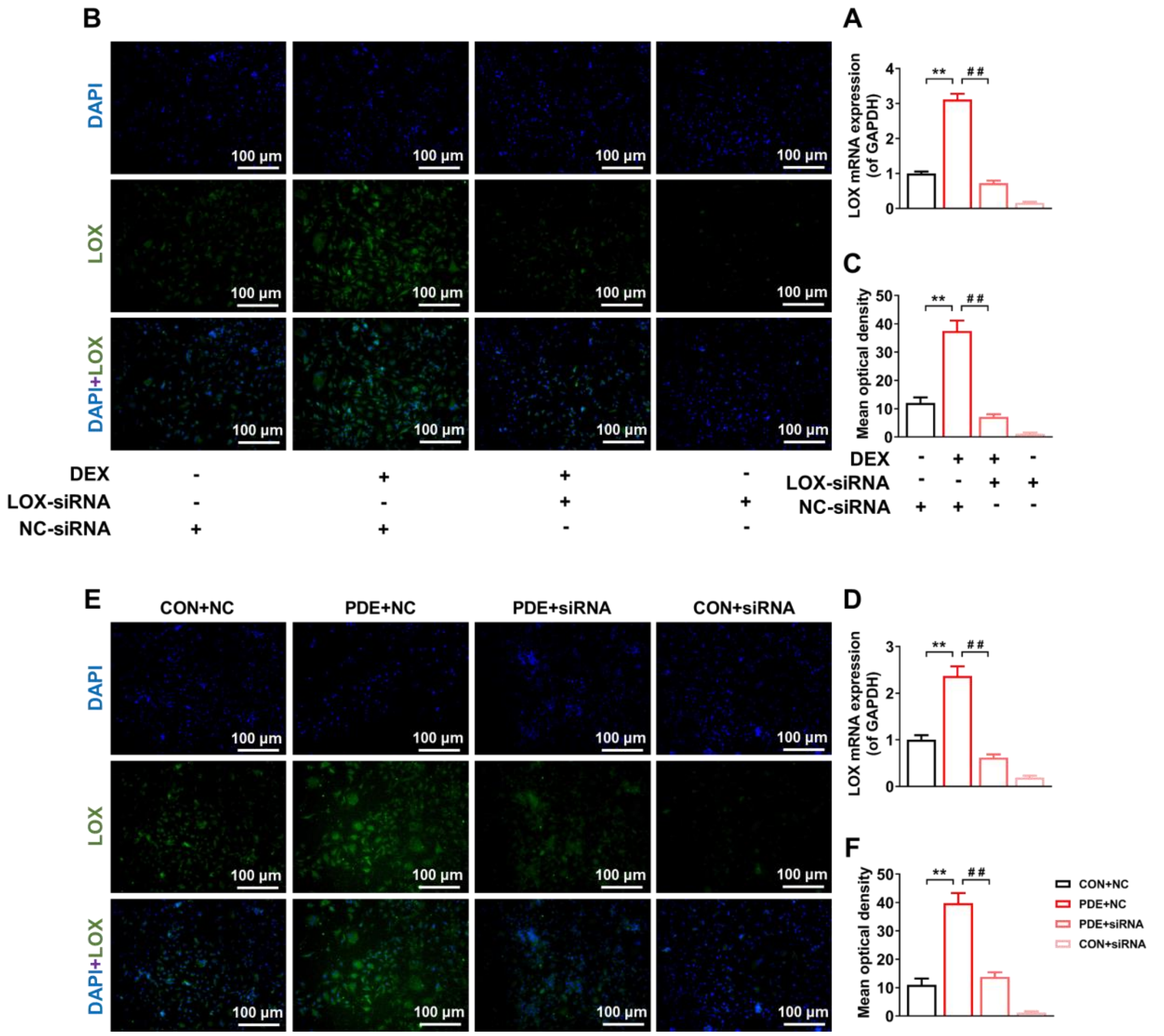

Fig S2. The experiments of the knockdown efficiency of LOX-siRNA on BMMs. (A, D): LOX mRNA expression in osteoclasts. (B, E): Representative images of LOX immunohistochemical-paraffin staining with DAPI in osteoclasts. (C, F): Quantification analyses of LOX protein mean optical density. Mean ± S.E.M., n=6 per group for mRNA expression, n=3 per group for LOX immunohistochemical-paraffin. **P<0.01 vs. NC-siRNA in vitro or CON+NC in vivo. ##P<0.01 vs. DEX+NC-siRNA in vitro or PDE+NC in vivo. DEX: dexamethasone; LOX: lysyl oxidase; NC: negative control; CON: control; PDE: prenatal dexamethasone exposure.

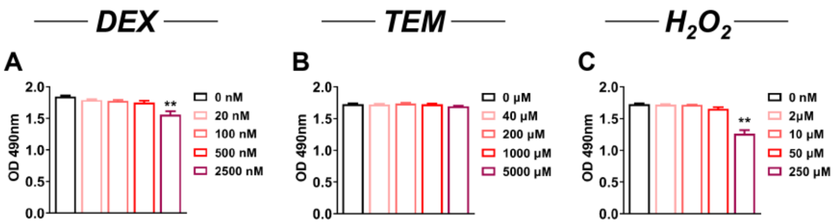

Fig S3. Cell viability experiments on BMMs. (A) The cytotoxic effect of DEX on BMMs at different concentrations was measured by the MTS assay. (B) The cytotoxic effect of TEM on BMMs at different concentrations was measured by the MTS assay. (C) The cytotoxic effect of H_2O_2 on BMMs at different concentrations was measured by the MTS assay. Mean ± S.E.M., n=6 per group. $^{**}P<0.01$ *vs.* 0 group. DEX: dexamethasone; TEM: tempol; BMMs: bone marrow-derived macrophages; MTS: 3-(4,5-dimethyltiazol-2-yl)-5-(3-carboxymethoxyphenyl)-2-(4-sulfophenyl)-2H-tetrazolium.

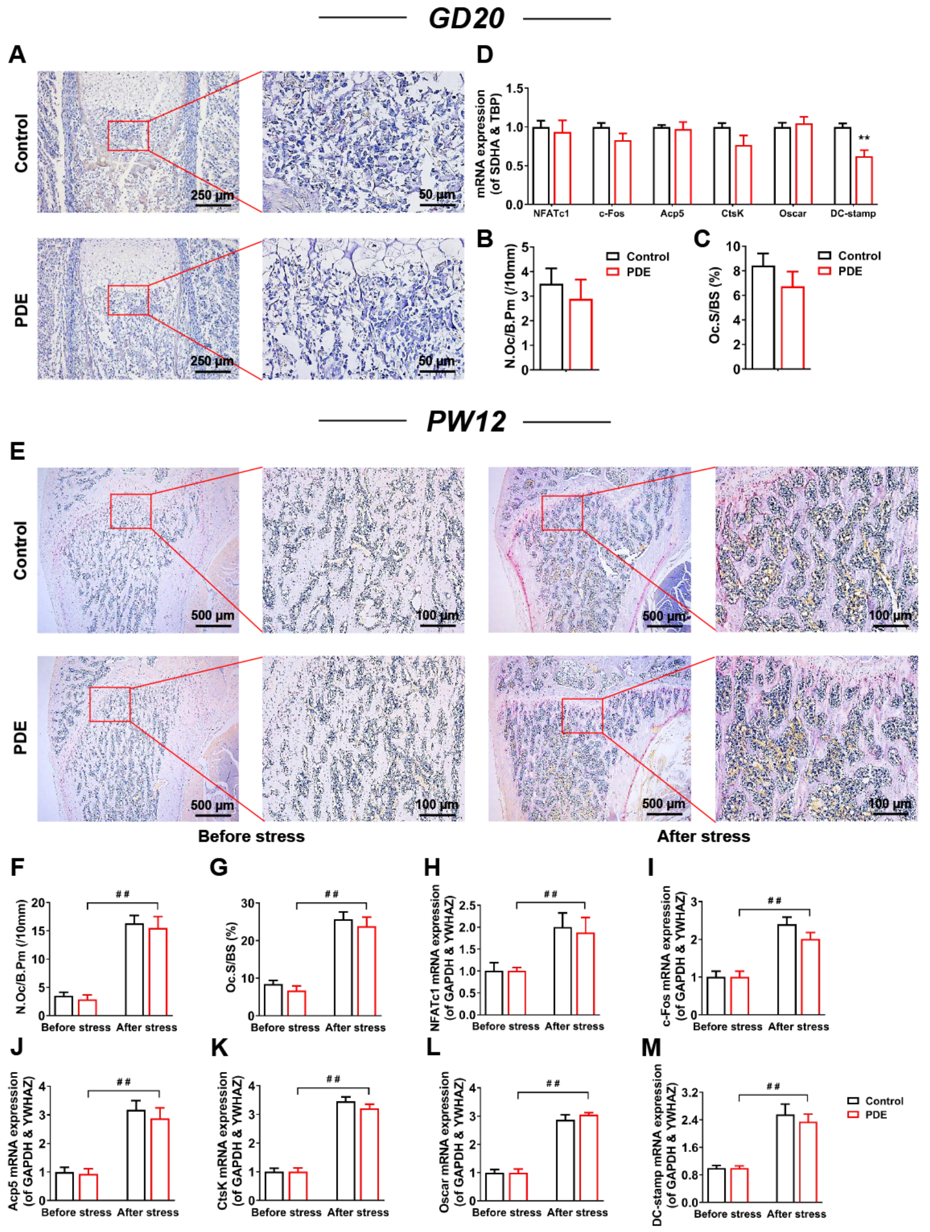

Fig S4. PDE induced no significant change of osteoclast function in male offspring rats. (A, E): Representative images of TRAP staining in decalcified bone sections of fetal rats and adult offspring rats. (B, F): Quantification analyses of N.Oc/B.Pm. (C, G): Quantification analyses of Oc.S/BS. (D): NFATc1, c-Fos, Acp5, CtsK, Oscar and DC-stamp mRNA expression in bone tissue of fetal rats. (H-M): NFATc1, c-Fos, Acp5, CtsK, Oscar and DC-stamp mRNA expression in bone tissue of adult offspring rats. Mean ± S.E.M., n=3 per group for TRAP staining, n=8 per group for mRNA expression. ***P*<0.01 *vs.* Control. $^{\#\#}$*P*<0.01 *vs.* PDE. PDE: prenatal dexamethasone exposure; TRAP: tartrate resistant acid phosphatase; N.Oc/B.Pm: osteoclast number per bone perimeter; Oc.S/BS: osteoclast surface per bone surface; NFATc1: nuclear factor of active T cells 1; c-Fos: protooncogene c-Fos; Acp5: acid phosphatase 5; CtsK: cathepsin K; Oscar: osteoclast-associated receptor; DC-stamp: dendritic cell specific transmembrane protein; SDHA: succinate dehydrogenase; TBP: TATA box binding protein; GAPDH: glyceraldehyde-3-phosphate dehydrogenase; YWHAZ: tyrosine 3-monooxygenase/tryptophan 5-monooxygenase activation protein, zeta.

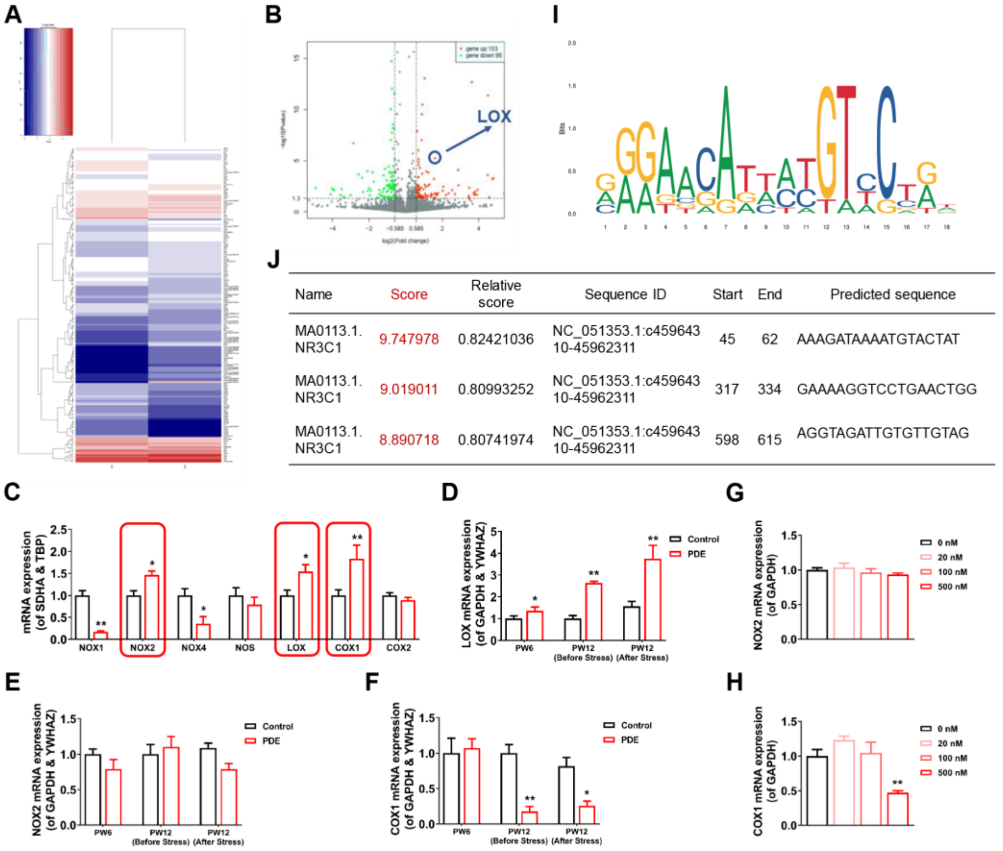

Name	Score	Relative score	Sequence ID	Start	End	Predicted sequence
MA0113.1. NR3C1	9.747978	0.82421036	NC_051353.1:c459643 10-45962311	45	62	AAAGATAAAATGTACTAT
MA0113.1. NR3C1	9.019011	0.80993252	NC_051353.1:c459643 10-45962311	317	334	GAAAAGGTCCTGAACTGG
MA0113.1. NR3C1	8.890718	0.80741974	NC_051353.1:c459643 10-45962311	598	615	AGGTAGATTGTGTTGTAG

Fig S5. Transcriptome sequencing results of female fetal rat long bone tissue and related experiments. (A, B): Transcriptome sequencing assays were performed in bone tissue of fetal rats. (C): ROS synthesis-related genes mRNA expression in bone tissue of fetal rats. (D): LOX mRNA expression in bone tissue of offspring rats. (E, F): NOX2 and COX1 mRNA expression in bone tissue of offspring rats. (G, H): NOX2 and COX1 mRNA expression in osteoclasts treated with different concentrations of DEX. (I, J): Predicted binding site of GR to LOX promoter region. Mean ± S.E.M., n=8 per group for mRNA expression *in vivo*, n=6 per group for mRNA expression *in vitro*. $^{*}P$<0.05, $^{**}P$<0.01 *vs.* Control *in vivo* or 0 nM DEX *in vitro*. LOX: lysyl oxidase; PDE: prenatal dexamethasone exposure; NOX: NADPH oxidases; NOS: NO synthase; COX: cyclooxygenase; SDHA: succinate dehydrogenase; TBP: TATA box binding protein; GAPDH: glyceraldehyde-3-phosphate dehydrogenase; YWHAZ: tyrosine 3-monooxygenase/tryptophan 5-monooxygenase activation protein, zeta; DEX: dexamethasone.

Table S1. The LOX siRNA sequences used in BMMs transfection experiment.

Genes	Forward sequences (5’-3’)	Reverse sequences (5’-3’)
LOX siRNA-1	GCACAGUUGUCAUCAACAUTT	AUGUUGAUGACAACUGUGCCA
LOX siRNA-2	GGGCAGAUGUCAGAGAUUAAT	UAAUCUCUGACAUCUGCCCAG
LOX siRNA-3	GAAUCUGACUAUACCAACATT	UGUUGGUAUAGUCAGAUUCAG

LOX: lysyl oxidase; BMMs: bone marrow-derived macrophages.

Table S2. Rat oligonucleotide primers and reaction conditions used in RT-qPCR.

Genes	Forward primers (5’-3’)	Reverse primers (5’-3’)	Annealing (°C)
NFATc1	CCGTTGCTTCCAGAAAATAACA	TGTGGGATGTGAACTCGGAA	60
c-Fos	AGCTCCCACCAGTGTCTACC	TCACCGTGGGGATAAAGTTGG	60
Acp5	CAAAGAGATCGCCAGAACCG	GAGACGTTGCCAAGGTGATC	60
CstK	CAGAGGCCACAACTCTCAGAA	GTGTCCATCGATGCAAGCTT	60
Oscar	CGATTGGCACAGCAGGCG	AAGACACATGAAGGAAATAGAG	60
DC-stamp	TCTGCTGTATCGGCTCATTTC	GCTCACGGGTCATCTTCATT	60
GR	CACCCATGACCCTGTCAGTC	AAAGCCTCCCTCTGCTAACC	58
ERβ	GGTCTGGGTGATTGCGAAGA	AGAAGCATCAGGAGGTTGGC	60
Dnmt1	AGCAAGGTCAAGGTCATCTA	GGCATTTCTTTTTGTCTCAG	60
Dnmt3a	ATCTACGAAGTCCTCCAGGT	ACATGTCGGTGTAAACTTCC	60
Dnmt3b	TCTGATGTCACTAACAACAA	GTGCAGTAGGACTGATAGCC	60
Tet1	TCACCAGAGGATCTTGGTGCTA	GCAGCTATTACCAGAGGTACTG	60
Tet2	GGAGGGATAAAACGCACAGTCA	GTTCCGTGTTGGGAAAGCATCT	60
Tet3	AGTTGATGGACCTGTTCCAGGT	GACTCATCTCACGGTTGAAGGT	60
NOX1	TCTGTTCTCTCCAGCCTATT	GGCCAGCAATACTGGTAAA	60
NOX2	CCAGTGAAGATGTGTTCAGCT	GCACAGCCAGTAGAAGTAGAT	60
NOX4	GTCCCAGTGTATCAGCATTAG	CTGTCCAGTCTCCTACTACTT	60
NOS	CACATGCAGAATGAGTACCG	GTTCAATATCTCCTGGTGGAAC	60
LOX	CAGGGTACTGCTACGATTTC	TCATCCATGCTGTGGTAATG	60
COX1	GGATCTGGGAGTTTGTGAATG	GTCATGTGCTGTGTTGTAGG	60
COX2	GTTGACGTCCAGATCACATT	TGGTGTAGTAGGAGAGGTTG	60
GAPDH	GCCTCCAAGGAGTAAGAAAC	GTCTGGGATGGAATTGTGAG	60
YWHAZ	TTGAGCAGAAGACGGAAGGT	CCTCAGCCAAGTAGCGGTAG	60
SDHA	CGAGATCCGTGAAGGAAGAG	GCCCATGTTGTAATGCACAG	60
TBP	TATAATCCCAAGCGGTTTGC	CAGCCTTATGGGGAACTTCA	60

RT-qPCR: real-time quantitative polymerase chain reaction; NFATc1: nuclear factor of active T cells 1; c-Fos: protooncogene c-Fos; Acp5: acid phosphatase 5; CtsK: cathepsin K; Oscar: osteoclast-associated receptor; DC-stamp: dendritic cell specific transmembrane protein; GR: glucocorticoid receptor; ERβ: estrogen receptor β; Dnmt: DNA methyltransferase; Tet: ten-eleven translocation

protein; NOX: NADPH oxidases; NOS: NO synthase; LOX: lysyl oxidase; COX: cyclooxygenase; GAPDH: glyceraldehyde-3-phosphate dehydrogenase; YWHAZ: tyrosine 3-monooxygenase/tryptophan 5-monooxygenase activation protein, zeta; SDHA: succinate dehydrogenase; TBP: TATA box binding protein.

Table S3. Rat oligonucleotide primers and reaction conditions used in ChIP-PCR.

Genes	Forward primers (5'-3')	Reverse primers (5'-3')	Annealing (°C)
LOX	ACTTCCTGGACATGATGGCA	CAAGCATACTGTCCATGGCC	60

ChIP-PCR: chromatin immunoprecipitation polymerase chain reaction; LOX: lysyl oxidase.
